# Kaposi's Sarcoma Herpesvirus MicroRNAs Induce Metabolic Transformation of Infected Cells

**DOI:** 10.1371/journal.ppat.1004400

**Published:** 2014-09-25

**Authors:** Ohad Yogev, Dimitris Lagos, Tariq Enver, Chris Boshoff

**Affiliations:** 1 UCL Cancer Institute, Research Department of Cancer Biology, University College London, London, United Kingdom; 2 Centre for Immunology and Infection, Department of Biology and Hull York Medical School, University of York, York, United Kingdom; Duke University Medical Center, United States of America

## Abstract

Altered cell metabolism is inherently connected with pathological conditions including cancer and viral infections. Kaposi's sarcoma-associated herpesvirus (KSHV) is the etiological agent of Kaposi's sarcoma (KS). KS tumour cells display features of lymphatic endothelial differentiation and in their vast majority are latently infected with KSHV, while a small number are lytically infected, producing virions. Latently infected cells express only a subset of viral genes, mainly located within the latency-associated region, among them 12 microRNAs. Notably, the metabolic properties of KSHV-infected cells closely resemble the metabolic hallmarks of cancer cells. However, how and why KSHV alters host cell metabolism remains poorly understood. Here, we investigated the effect of KSHV infection on the metabolic profile of primary dermal microvascular lymphatic endothelial cells (LEC) and the functional relevance of this effect. We found that the KSHV microRNAs within the oncogenic cluster collaborate to decrease mitochondria biogenesis and to induce aerobic glycolysis in infected cells. KSHV microRNAs expression decreases oxygen consumption, increase lactate secretion and glucose uptake, stabilize HIF1α and decreases mitochondria copy number. Importantly this metabolic shift is important for latency maintenance and provides a growth advantage. Mechanistically we show that KSHV alters host cell energy metabolism through microRNA-mediated down regulation of EGLN2 and HSPA9. Our data suggest that the KSHV microRNAs induce a metabolic transformation by concurrent regulation of two independent pathways; transcriptional reprograming via HIF1 activation and reduction of mitochondria biogenesis through down regulation of the mitochondrial import machinery. These findings implicate viral microRNAs in the regulation of the cellular metabolism and highlight new potential avenues to inhibit viral latency.

## Author Summary

Kaposi's sarcoma (KS) is the most common cancer in HIV-infected untreated individuals. Kaposi's sarcoma-associated herpesvirus (KSHV) is the infectious cause of this neoplasm. The discovery of KSHV and its oncogenic enigmas has enlightened many fields of tumor biology and viral oncogenesis. The metabolic properties of KS significantly differ from those of normal cells and resemble cancer cells in general, but the mechanisms employed by KSHV to alter host cell metabolism are poorly understood. Our work demonstrates that KSHV microRNAs can alter cell metabolism through coherent control of independent pathways, a key feature of microRNA-mediated control of cellular functions. This provides a fresh perspective for how microRNA-encoding pathogens shape a cell's metabolism to create an optimal environment for their survival and/or replication. Indeed, we show that, in the case of KSHV, viral microRNA-driven regulation of metabolism is important for viral latency. These findings will evoke new and exciting approaches to prevent KSHV from establishing latency and later on KS.

## Introduction

Viruses are the etiological agents in approximately 12% of human cancers. Most of these cancers can be attributed to infections by human papillomavirus (HPV), hepatitis B virus (HBV), hepatitis C virus (HCV), Epstein-Barr virus (EBV), and Kaposi's sarcoma-associated herpesvirus (KSHV) [1], [2]. KSHV is the etiological agent of Kaposi's sarcoma (KS) and it is also causally linked to primary effusion lymphoma (PEL) and a subset of multicentric Castleman's disease [3]–[5]. KSHV, like other herpesviruses, can enter a latent viral program after multiple rounds of replication and infection of new target cells [6].

In traditional models of herpesvirus-induced tumorigenesis, latency has the primary role in oncogenesis, promoting cell proliferation and impairing apoptosis. The lytic cycle is not considered to directly contribute to oncogenesis, but plays an earlier role by allowing viruses to disseminate in the host and to infect the target cells [6]. Consistently, in KS tumors and in PEL, the majority of cells are latently infected and express only a subset of viral genes located within the latency-associated region. This includes the viral-encoded cyclin (*vCyclin*), FLIP (*vFLIP*), latency-associated nuclear antigen (*LANA*) and 12 microRNAs (miRNAs) (to express 17 mature miRNAs) [7]–[12].

miRNAs are regulatory RNAs expressed by animals, plants and some viruses [13], [14]. They are synthesized as precursors that fold into imperfect double strand RNA hairpins. This structure is cleaved in two steps, which are catalysed by Drosha and the endoribonuclease Dicer RNaseIII nucleases. This process results in a ∼22 base pair miRNA duplex. One strand of this duplex can be incorporated into the RNA induced silencing complex (RISC) as a mature miRNA. Within RISC, miRNAs are bound by Argonaute (Ago) proteins and induce repression of mRNAs bearing sequences with partial complementary to the miRNA.

Ten out of twelve of the KSHV miRNAs (miR-K12-1 to 9, and 11) are located within the intron of K12 and are expressed as a cluster [15]. Although the expression levels of these miRNAs varies between different cells lines and KS samples, it has been consistently shown that all 10 miRNAs are expressed together in latently infected cells [16]–[19].

Recent studies have suggested that approximately half of all human miRNAs are expressed and function as clusters; targeting the same mRNA or different mRNAs involved in the same pathway [20]–[22]. Recent cross-linking immunoprecipitation (CLIP) experiments in PEL cell lines have indicated that this also might apply to the KSHV miRNAs [16], [17]. This suggests that these miRNAs could have developed in the course of viral evolution to function as a cluster during latent infection.

The characteristic metabolic hallmark of tumor metabolism is aerobic glycolysis; In contrast to normal differentiated cells, which rely primary on mitochondrial oxidative phosphorylation to generate energy, most cancer cells instead rely on aerobic glycolysis, a phenomenon termed as the Warburg effect. Cancer cells metabolism is a result of the modulation of intracellular signaling pathways that are disrupted by mutated oncogenes and tumor suppressors. Moreover alteration in cell metabolism may trigger tumorigenesis [23]. Viruses do not inherently have their own metabolic output. However, upon infection, viruses dramatically alter the metabolism of the host cell. This can provide substrates necessary for viral replication and is also likely to be important for pathogenesis. Human cytomegalovirus (HCMV), HCV, human immunodeficiency virus (HIV), herpes simplex virus (HSV) and KSHV have all been shown to alter cell host metabolism [24]–[30].

Mitochondria are unique and complex organelles that perform essential functions in many aspects of cell biology. The dominant function of mitochondria is the production of more than 90% of the cell's energy in the form of ATP through oxidative phosphorylation (OXPHOS). The oxidative phosphorylation system consists of four multimeric complexes, coenzyme Q and cytochrome c forming the mitochondrial respiratory chain (I–IV), which transfer electrons from reducing equivalents to water, creating a proton gradient across the inner mitochondrial membrane, which is used by a fifth complex, the F1F0 ATPase, to drive the synthesis of ATP [31]. In addition to their role in energy metabolism, mitochondria also perform various other functions, which make them absolutely indispensable to the cell. Among these, mitochondria are implicated in apoptosis, the regulation of various metabolism pathways, and signal transduction of antiviral responses [32]–[34]. This makes them a target of almost all invading pathogens, including viruses [34]. Although mitochondria possess a separate and independent genome, most of the mitochondrial proteins are encoded in the nucleus, translated in the cytosol and imported into mitochondria [35]. Therefore, mitochondrial genes are exposed to post-transcriptional regulation by miRNAs.

The hypoxia-inducible factor 1 alpha (HIF1α) is one of the master regulators of cell metabolism and was shown to inhibit mitochondrial biogenesis [36]. Under normoxic conditions, HIF1α is hydroxylated by the three HIF prolyl hydroxylases (HPHs) EGLN1-3 (PHD1-3) and this marks it for proteosomal degradation [37], [38]. Under hypoxic condition, the HPHs are suppressed, and consequently HIF1α is stabilized [38]. Stabilized HIF1heterodimerizes with HIF1β and binds to the hypoxia response elements (HREs) in numerous target genes to activate their transcription [39]. HIF1 is known to mediate an active switch from oxidative phosphorylation to glycolytic metabolism [40]–[45].

Although the KSHV genome encodes 12 miRNAs, up to now, only a handful of their targets have been confirmed [46]. Here we show that the KSHV miRNAs function as a cluster to induce metabolic transformation during latent infection from oxidative phosphorylation to aerobic glycolysis. We identified and confirmed two specific targets for these miRNAs that suggest a possible mechanism for this metabolic transformation: the HIF prolyl hydroxylase EGLN2 and the heat shock protein HSPA9. We also show that this metabolic conversion contributes to the growth of latently infected cells under hypoxia and promotes latency maintenance.

## Results

### KSHV miRNAs induce a metabolic transformation in LEC

KSHV was shown to induce the Warburg effect in latently infected endothelial cells, including lymphatic endothelial cells (LEC), and in KS [24], [25]. However, the mechanisms behind these alterations are not clear, and it is not known whether KSHV has a direct effect on mitochondrial function.

During latency KSHV expresses only a subset of genes including *vcyclin*, *vFLIP*, *LANA* and the miRNA cluster. This suggests that one or more of these genes are responsible for changing cellular glucose metabolism.

In order to test this hypothesis, we examined the effect of each of the latent proteins and the miRNA cluster on glucose metabolism. Metabolic output is known to vary between different cell types and miRNA function is affected by cell context. Therefore, we focused on primary dermal microvascular LEC, which are believed to be the progenitor cells for KS [47]–[49].

We used lentiviruses to express each of the latent proteins or the miRNA cluster in LEC (Figure S1A–E). When we expressed the miRNA cluster, all the individual miRNAs are expressed and intriguingly the expression level of each one of them is similar to its expression in KSHV infected LEC (KLEC) (Figure S1E–F). Since over expression of exogenous miRNAs in cells can have non-specific effects, we created a mutated version of the miRK12-3, which is one of the most expressed among the KSHV miRNAs (Figure S1E and F), and used it as a control for expression of the KSHV miRNA cluster. There are two obvious characteristics that indicate a shift in glucose metabolism from oxidative phosphorylation (OXPHOS) to aerobic glycolysis: reduced oxygen consumption and acidification of growth media due to secretion of the glycolysis product lactate. We first tested oxygen consumption rate using the Seahorse XF24 analyzer. The Seahorse Extracellular Flux Analyzer determines oxygen consumption rate (OCR), and extracellular acidification rate (ECAR), in order to assess cellular functions such as oxidative phosphorylation and glycolysis.

**Figure 1:**
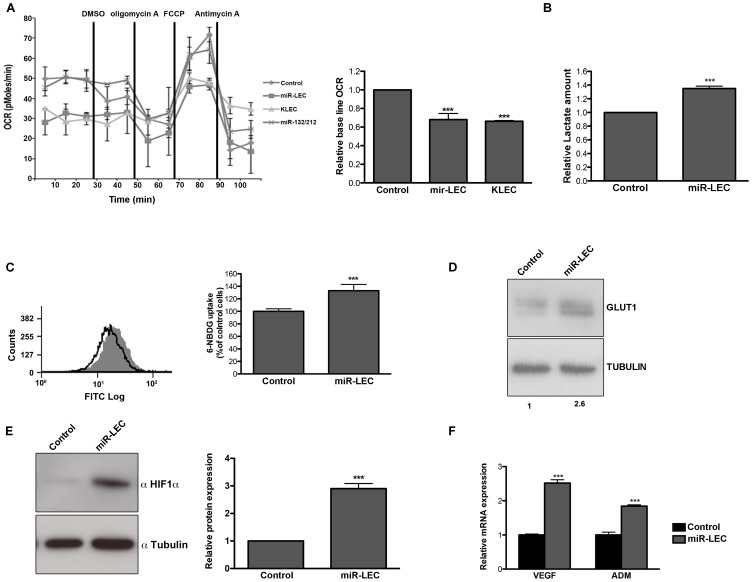
KSHV miRNA cluster induces aerobic glycolysis and stabilizes HIF 1 alpha. **A.** Oxygen consumption rate (OCR) in cells expressing a non-targeting control vector or the KSHV miRNA cluster was measured using the Seahorse XF24 Analyser. Cells were seeded at a density of 4×10^4^ cells per well and the assay was performed according to the manufacturer's Mito stress protocol. Uncoupled, maximal and non-mitochondrial respiration was determined after the addition of 5 µM oligomycin, 1 µM carbonyl cyanide 4-(trifluoromethoxy)phenylhydrazone (FCCP) and 2 µM antimycin-A. The bar graph presents the average base line OCR in 3 independent experiments relative to non-targeting control OCR (Mean+SEM, n = 3). **B.** Lactate levels in the control and miR-LEC culture media. Equal numbers of cells were grown for 24 hours and lactate levels in the media were measured using the MBL Lactate Colorimetric assay kit. The bar graph presents the average ratio between control and miR-LEC from 3 independent experiments (Mean+SEM, n = 3). **C.** Glucose uptake into control and miR-LEC cell. Control and miR-LEC were incubated with 30 µM of the fluorescent glucose analogue 6-NBDG for 20 minutes prior to analysis by fluorescence-activated cell sorter (FACS). The histogram displays one representative experiment with control shown in black and miR-LEC shown in grey. The bar graph presents the average ratio between the control and miR-LEC from 3 independent experiments (Mean+SEM, n = 3). **D.** GLUT1 protein expression, as measured by Western blotting, in control or miR-LEC. Values indicate the relative signal of the GLUT1 antibody normalized to α-tubulin as measured using the Odyssey. **E.** HIF1α protein expression, as measured by Western blotting, in LEC expressing the viral miRNA cluster or the control vector. The bars show relative values of HIF1α antibody intensity normalized to α-tubulin (Mean+SEM, n = 3). **F.** Expression of the HIF1α target genes *VEGF* and *ADM*. mRNA levels were determined by quantitative real-time PCR (qRT-PCR). Tubulin beta (*TUBB*) levels were used for normalization. In all panels statistical significance denoted by *P<.05; **P<.01; ***P<.001.

While expression of LANA, vcyclin or vFLIP did not have a significant effect on oxygen consumption (Figure S1G), expression of the KSHV miRNA cluster (miR-LEC) reduced base line oxygen consumption to a similar level to this in KLEC (Figure 1A). In concordance with this finding we also observed a ∼33% increase of secreted lactate from miR-LEC cells when lactate levels were measured directly from the growth media (Figure 1B).

Another known marker of aerobic glycolysis is increased glucose uptake. We monitored glucose uptake into cells by measuring the uptake of the fluorescently labeled deoxyglucose analog 6-NBDG and found increased uptake of ∼30% in cells expressing miR-LEC (Figure 1C), but not in those expressing latent proteins (Figure S1J). We also found that the glucose transporter GLUT1 is overexpressed by over 2-fold in these cells (Figure 1D); a second indicator of increased glucose uptake.

KSHV express additional two miRNAs out of the cluster; miR-K10-10 and miR-K12-12. Although these miRNAs expressed in a relative high levels in KLEC (Figure S1F) we could not find any significant change in oxygen consumption, lactate secretion or glucose uptake when expressing these miRNAs in miR-LEC (Figure S1 H–I and K).

To further rule out unspecific effects caused by expression of a miRNA cluster we used miR-132/212 cluster as a second control. As shown in figure 1A and figures S1L–M, expression of this cluster does not affect oxygen consumption or glucose uptake. Taken together, these results suggest that the KSHV miRNAs have the ability to shift host cell metabolism toward aerobic glycolysis.

The hypoxia-induced factor alpha (HIF1α) is a known regulator of glucose metabolism [50], [51] and can mediate the Warburg effect in cancer cells [52].

KSHV was also shown to activate HIF1 and HIF2 alpha during latency [53]. We therefore tested whether the miRNA-induced alteration in cell metabolism is related to HIF1α expression and activity. As shown in figure 1E, in miR-LEC HIF1α protein is overexpressed by ∼3 fold compared to the control cells. Correspondingly, we found increased expression of two known targets genes of HIF1α: *VEGFA* (vascular endothelial growth factor A) and *ADM* (adrenomedullin) (Figure 1F). This concurred with increased HIF-1 transcriptional activity as shown by increased luciferase activity when the miRNAs were expressed together with a HIF-1 luciferase-reporter assay [54] (Figure S1N).

Taken together these results suggest that expression of the miRNA cluster in LEC is sufficient to change glucose metabolism from oxidative phosphorylation to aerobic glycolysis and this might occur due to HIF1α stabilization.

### KSHV miRNAs reduce mitochondrial number

**Figure 2:**
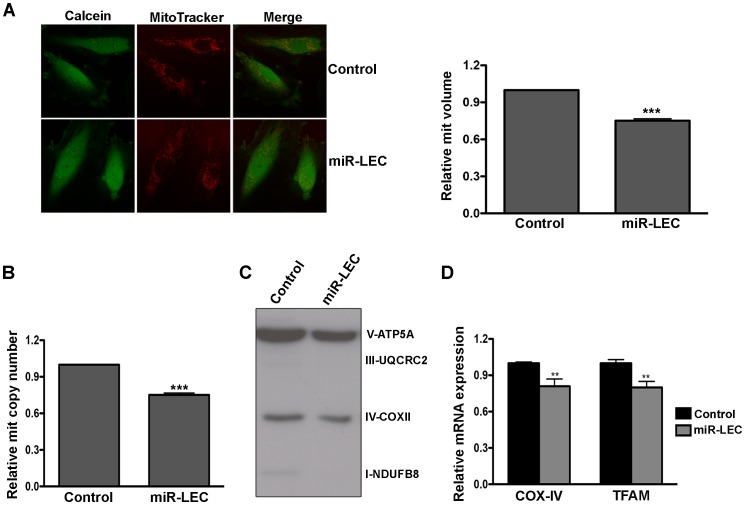
Expression of the KSHV miRNA cluster reduces mitochondrial biogenesis. **A.** Mitochondrial volume in miR-LEC. Cells were loaded with 5 µM Calcein-AM and 5 nM MitoTracker Deep Red FM and Z-series of images were. Maximal projections of images were used to quantify the area of green (Calcein) and red (MitoTracker Deep Red) signals as previously described [88]. Representative single-plane images of the mitochondrial structure are shown on the left panel. The bar graph on the right presents the average relative mitochondrial volume in miR-LEC compared to control cells (Mean±SEM, n = 5). **B.** Mitochondrial DNA (mtDNA) copy number in cells expressing the viral miRNA cluster relative to control cells. qPCR was carried out as described in [89]. **C.** Expression levels of the 5 OXPHOS complexes as measured by Western blotting analysis using the MitoProfile Total OXPHOS Human WB Antibody Cocktail in miR-LEC and the control cells. In all panels statistical significance denoted by *P<.05; **P<.01; ***P<.001. **D.** Expression of *COXIV* and *TFAM* in cells expressing the KSHV miRNA cluster relative to control cells. mRNA levels were determined by qRT-PCR. TUBB levels were used for normalization.

Mitochondria are key players in normal glucose metabolism in aerobic conditions, and as part of the Warburg effect, many cancer types show altered mitochondrial activity [55]. Having found that expression of KSHV miRNAs reduces oxidative phosphorylation, we next tested their effect on mitochondrial function. As an initial assessment of mitochondrial function, we loaded cells with MitoTracker together with Calcein AM. MitoTracker is a fluorescent dye that labels mitochondria within live cells utilizing the mitochondrial membrane potential. It therefore allowed us to calculate mitochondrial volume (MitoTracker staining) relative to total cell volume (Calcein staining). Although we could not detect any change in mitochondrial structure, we did find a significant decrease in mitochondria volume in miR-LEC (Figure 2A). Calculation of mitochondria volume in KLEC showed again similar reduction to this in miR-LEC while the miR-132/212 did not have any effect (Figure S2A). When we tested the expression of miR-K12-10 and miR-K12-12 in miR-LEC we found again no significant different (Figure S2B). Since mitochondria have their own independent genome, one can calculate their number by qPCR analysis using specific primers for the mitochondrial genome [56]. Total DNA (mitochondrial and genomic), extracted from miR-LEC and control cells, was used as template for this analysis. This showed that while the control cells had between 50–60 copies of the mitochondrial genome, in miR-LEC there was a 25% decrease in mitochondrial DNA (Figure 2B). Measuring the expression of different OXPHOS complexes using an antibody cocktail, showed a similar decrease in their expression in miR-LEC (Figure 2C and Figure S2C–D). Next, we examined whether this reduction in mitochondrial number is due to reduced mitochondrial biogenesis. For this we measured the mRNA levels of *COX-IV* (Cytochrome c oxidase subunit IV) and *TFAM* (mitochondrial transcription factor A) as expression of these genes has been shown to correlate with mitochondrial biogenesis [57], [58]. Using qRT-PCR we found that expression levels of both genes are significantly decreased by ∼20% in miR-LEC (Figure 2D).

Overall these findings suggest that expression of the miRNA cluster leads to decreased mitochondrial number and activity.

### The KSHV miRNA cluster regulates EGLN2 and HSPA9

miRNAs act to alter the translation and mRNA stability of specific genes and consequently they regulate the cellular pathways those genes control. Therefore, we investigated potential genes that are regulated by the KSHV miRNAs. For this we combined data from three prediction algorithms (PITA [59], miRanda [60] and TargetScan [61]), together with published data for the KSHV miRNAs targetome from two different CLIP experiments [16], [17]. This allowed us to identify a number of potential genes that might be regulated by these miRNAs resulting in altered cellular glucose metabolism. All three different HIF prolyl hydroxylases (*EGLN1-3*) were predicted to be targets of the viral miRNAs. *EGLN1* was suggested to be targeted by miR-K12-2 and miR-K12-11 in both BC-1 and BC-3 PEL cell lines through a CLIP experiment [16], while *EGLN2* is predicted by the different algorithms to be targets by multiple miRNAs (miR-K12-2, miR-K12-3, miR-K12-6, miR-K12-7, miR-K12-8, miR-K12-9 and miR-K12-11).

It has been shown that in different cell types these three HIF prolyl hydroxylases (HPH) have different expression profiles and differential functions in the regulation of HIF1α [62]. We therefore tested the expression profile of these genes in LEC and found that *EGLN2* is the most expressed, whilst *EGLN3* is hardly expressed in LEC (Figure S3A). We next quantified the mRNA levels of these genes after expression of the viral miRNA cluster and found that only *EGLN2* and *EGLN3* were down regulated (Figure S3B). Taking these findings into consideration, we decided to focus only on regulation of *EGLN2* by the viral miRNAs for further study.

Interestingly, both previous CLIP studies identified in their pathway enrichment analysis genes that are involved in protein targeting and localization[16], [17]. In addition, the different prediction algorithms also predicted many of these genes to be regulated by the KSHV miRNAs. Since mitochondrial biogenesis depends on protein translocation, down regulation of proteins involved in this process might explain the reduced mitochondrial number and biogenesis induced by KSHV miRNAs. To investigate this potential regulatory axis, we chose 9 different genes, predicted to be regulated by the KSHV miRNAs, and tested their mRNA expression in miR-LEC (Figure S3C). We found that the mRNA of 5 of these genes was indeed down regulated in the presence of the KSHV miRNA cluster. In order to further examine this finding, we cloned the 3′UTR of these genes into a reporter plasmid, downstream of the luciferase coding sequence. As shown in Figure S3D, these 3′UTRs are indeed targeted by the KSHV miRNA cluster, resulting in decreased luciferase activity.

As the mitochondrial heat shock protein *HSPA9* was suggested to be a target of multiple viral miRNAs in both CLIP studies (miR-K12-3 and miR-K12-4) and by all three algorithms (miR-K12-3, 4, 5, 6, 7, 10 and 11), and it was shown to be the most down-regulated among the genes we tested, we focused on it for further studies. HSPA9 (mtHSP70, mortalin) is a central subunit of the matrix-exposed import motor playing a central role in the mitochondrial translocation system and is essential for efficient import and export of proteins [63], [64]. It was shown that it is crucial for viability and mitochondrial biogenesis [65], [66]. Therefore, its down regulation could contribute to the reduction of mitochondria in cells expressing the viral miRNAs.

**Figure 3:**
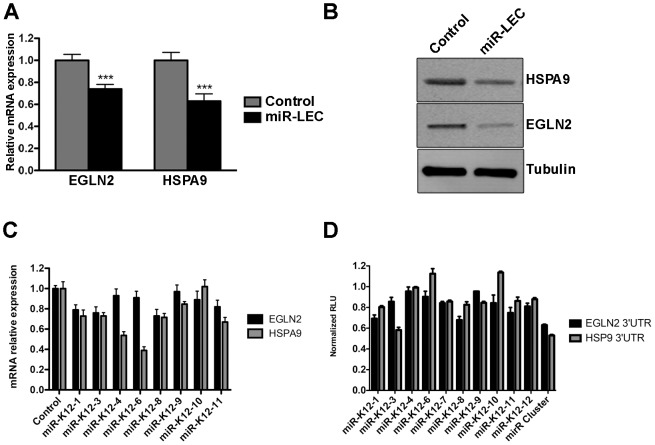
The KSHV miRNA cluster regulates EGLN2 and HSPA9. **A.** Relative mRNA levels of *EGLN2* and *HSPA9* in miR-LEC compared to control cells. mRNA levels were determined by qRT-PCR. TUBB levels were used for normalization. **B.** Protein levels of EGLN2 and HSPA9 in miR-LEC. Top right panel: protein expression, as measured by Western blotting, in miR-LEC and the control cells. **C.** Relative mRNA levels of *EGLN2* and *HSPA9* in LEC expressing the individual KSHV miRNAs. mRNA levels were determined by qRT-PCR. *TUBB* levels were used for normalization. **D.** Reporter assay indicating the sensitivity of the *EGLN2* or *HSPA9* 3′UTRs to targeting by the individual KSHV miRNAs. Firefly expression was normalized to Renilla expression to give the relative light units (RLU), which are shown relative to the non-targeting control. In all panels statistical significance denoted by *P<.05; **P<.01; ***P<.001.

To confirm that the KSHV miRNA cluster targets *EGLN2* and *HSPA9*, we measured their expression in miR-LEC. EGLN2 and HSPA9 mRNA and protein levels were significantly reduced in miR-LEC comparing to the controls cells (Figure 3A and B and Figure S3E). To confirm that these miRNAs specifically target the 3′UTR of these genes, we used a vector containing the luciferase coding sequence up-stream of their 3′UTRs. We expressed this construct in the presence of the miRNA cluster and observed ∼50% reduction in luciferase activity, while the control vector maintained luciferase activity in the presence of the miRNA cluster (Figure S3F). To confirm these genes are regulated also during KSHV infection, we tested their expression in KLEC and found both to be down regulated (Figure S3G).

Next, we tested our hypothesis that the viral miRNAs function as a cluster, and dissected the contribution of each individual miRNA to the regulation of *EGLN2* and *HSPA9*. For this we tested the mRNA levels of *EGLN2* and *HSPA9* in LEC upon expression of the separate miRNAs (Figure S3H and figure 3C). This showed that, as predicted, each one of these genes is targeted by more than one of the KSHV miRNAs (miR-K12-1, 3, 8, and 11 for *EGLN2* and miR-K12-1, 3, 4, 6, 8, 9 and 11 for *HSPA9*). Expressing luciferase vectors harboring the 3′UTRs of EGLN2 or HSPA9, together with the individual miRNAs, also showed that these 3′UTRs have target sites for more than one of the KSHV miRNAs (Figure 3D). Some miRNAs down regulated the mRNA levels of these genes but did not have any effect on luciferase activity (e.g. miR-K12-4 on HSPA9). These miRNAs may target predicted sites outside the 3′UTR or regulate ELGN2 and HSPA9 indirectly. Going back to the prediction algorithm PITA for these genes we found that indeed many of these miRNAs are also predicted to target these genes either in their 3′UTR or coding sequence (Table S1).

### The KSHV miRNA cluster can induce the Warburg effect through regulation of *EGLN2* and *HSPA9*

**Figure 4:**
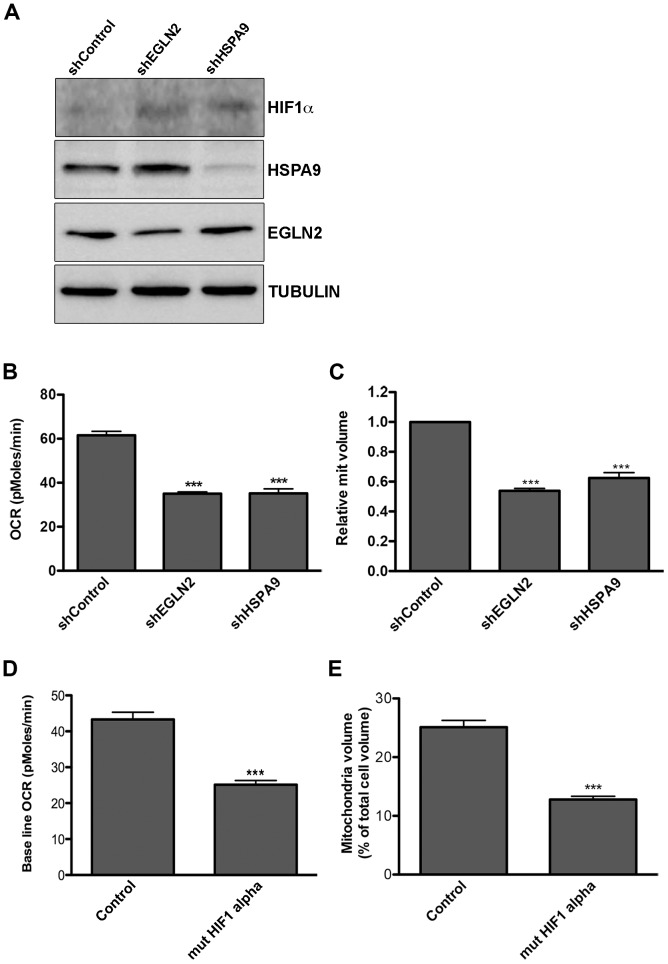
Down regulation of EGLN2 and HSPA9 phenocopies miRNA cluster expression. **A–C.** LEC were infected with specific hairpins for *EGLN2* and *HSPA9* and a non-targeting control (Open Biosystems) and analyzed for: A. Protein expression levels of HIF1α, HSPA9 and EGLN2, as measured by Western blotting; B. Base line OCR (as described in Figure 1A); C. Mitochondrial volume (as described in Figure 2A). **D–E.** LEC were infected with lentiviruses for HIF1α P402A/P564A (mut HIF1α) and analyzed for base line OCR (D) and mitochondrial volume (E).

To test whether the KSHV miRNAs trigger a shift in cell metabolism through down regulation of *EGLN2* and *HSPA9*, we used the human GIPZ shRNAmir lentiviral clones (Open Biosystems) to specifically suppress each of these genes. We down-regulated each one of these genes (Figure 4A and Figure S4A–C) and tested the effect on cellular metabolism in LEC. We found that knockdown of each of these genes led to stabilization of HIF1α, decreased oxygen consumption and reduced mitochondrial volume (Figure 4A–C). Because knockdown of EGLN2 and HSPA9 caused HIF1α stabilization, we next investigated whether the observed metabolic phenotype depends on HIF1α. We therefore expressed the HIF1α P402A/P564A stable mutant in LEC [67] (Figure S4D). As shown in Figure 4D–E, expression of this mutant resulted in a similar decrease in oxygen consumption and mitochondrial volume as that caused by *EGLN2* and *HSPA9* silencing, suggesting that HIF signaling is playing a role in this phenotype.

To further examine whether this metabolic transformation depends on EGLN2 and HSPA9, we next tested whether overexpression of these proteins could counteract the effect of the miRNA cluster. We achieved overexpression of both *EGLN2* and *HSPA9*, alongside viral miRNA expression, by using constructs that lacked 3′UTRs (Figure S5A). Expression of each miRNA-resistant gene reversed the effect of the viral miRNAs on its particular mRNA but not on the other gene's mRNA (Figure S5B), showing that the miRNA cluster down-regulates these genes independently.

**Figure 5:**
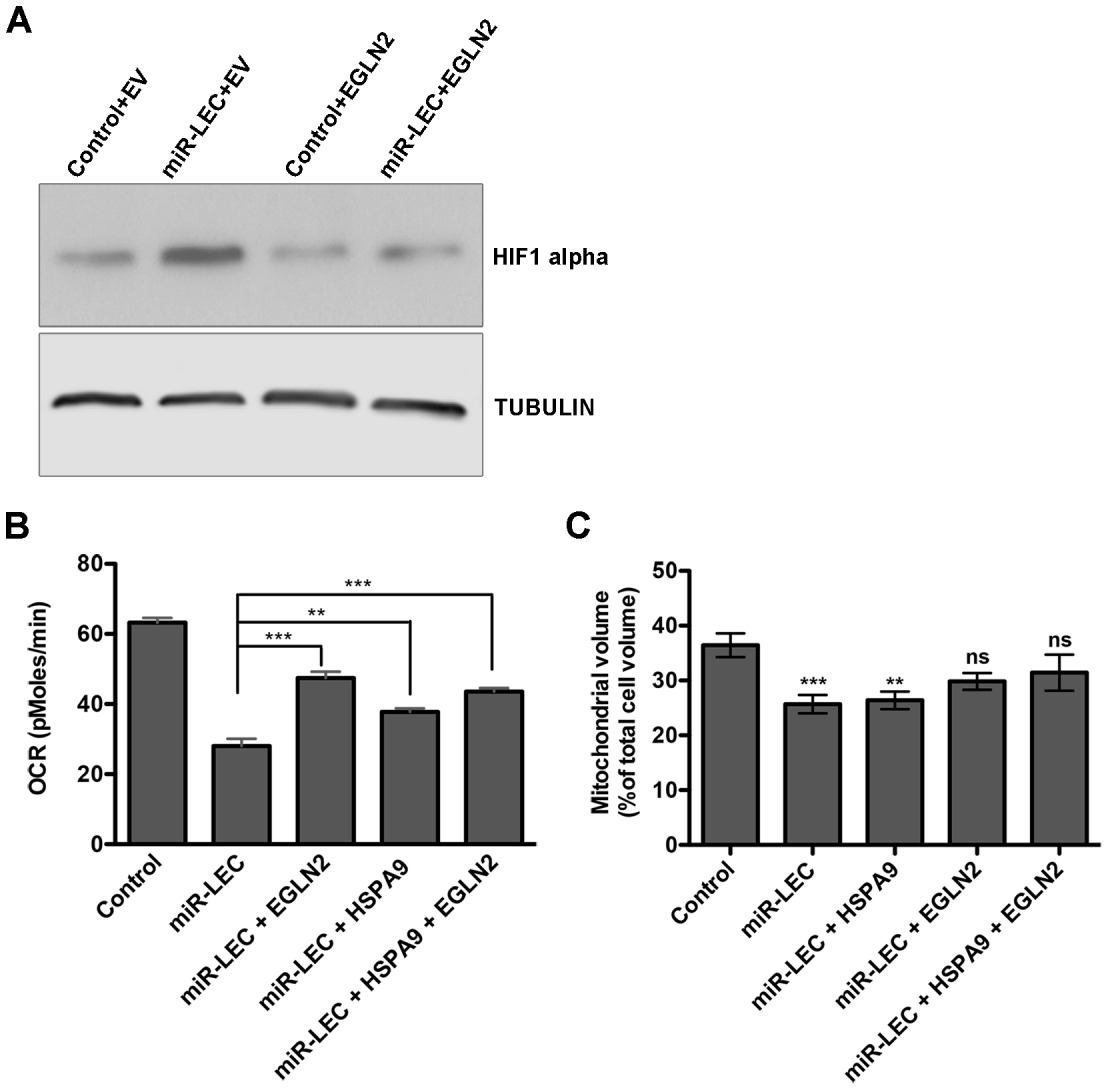
Overexpression of EGLN2 and HSPA9 partially rescues the miRNA cluster effect on glucose metabolism. **A.** HIF1α levels as measured by Western blotting in miR-LEC with or without overexpression of EGLN2 (EV = empty vector). **B.** Base line OCR was measured using the Seahorse XF24 Analyser in miR-LEC, with or without overexpression of EGLN2 and HSPA9 (as described in Figure 1A). **C.** Mitochondrial volume was measured as described in Figure 2A.

As expected, overexpression of EGLN2 prevented HIF1α stabilization by the miRNA cluster (Figure 5A). More importantly, we found that both EGLN2 and HSPA9 overexpression significantly reduced the effect of the viral miRNA cluster on oxygen consumption and mitochondrial volume (Figure 5B and C). Interestingly, EGLN2 up-regulation had a bigger effect on both oxygen consumption (Figure 5B), and mitochondrial volume (Figure 5C), when compared to HSPA9 overexpression. When both proteins were overexpressed together, they showed an additive effect. The fact that the miRNA cluster phenotype could not be fully reversed by overexpression of either EGLN2 alone, HSPA9 alone, or the combination of both proteins, suggests that although they are part of the miRNA-induced metabolic transformation mechanism, other genes also play a role.

### Inducing the Warburg effect provides a growth advantage in low oxygen conditions and is important for latency maintenance

**Figure 6:**
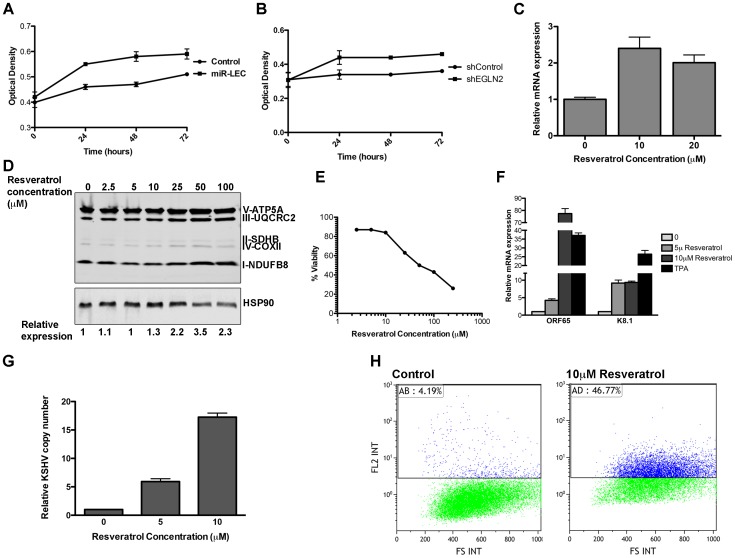
The miRNAs induced metabolic shift enhanced growth under hypoxia and is important for latency maintenance. **A–B.** 7500 cells expressing the KSHV miRNA cluster or the control vector (A) and cells expressing the non-targeting control or shEGLN2 (B) were plated in 96 well plates. Cells were fixed after 30 minutes, 24, 48 and 72 hours using 10% Trichloroacetic acid, stained with Sulforhodamine B, and plates were read at 564 nm. Optical density indicates the amount of proteins in the different wells. C. Expression of *COXIV* in BCBL1 cells treated with in indicated concentration of Resveratrol. mRNA levels were determined by qRT-PCR. TUBB levels were used for normalization. D. Expression of the 5 OXPHOS complexes, as measured by Western blotting analysis using the MitoProfile Total OXPHOS Human WB Antibody Cocktail in BCBL1 cells treated with the indicated concentration of Resveratrol. Values indicate the relative signal of the different antibodies normalized to HSP90 as measured using the ImageQuant software. E. BCBL1 cells were treated with the indicated concentration of Resveratrol for 48 hours. Viability was determined using the Muse Count & Viability Assay Kit on the Muse cell analyzer (Merck Millipore). F. Relative mRNA levels of the KSHV lytic genes *ORF65* and *K8.1* in BCBL1 cells treated with Resveratrol or TPA for 48 hours. mRNA levels were determined by qRT-PCR. TUBB levels were used for normalization. G. Relative KSHV DNA copy number in 293T cells infected using the growth media of BCBL1 cell treated with the incubated concentration of Resveratrol. H. LEC were infected with rKSHV.219 virus and selected as previously described [71]. Cells were treated with Resveratrol and analyzed by Flow cytometer after 72 hours. The numbers denote the percentage of RFP positive cells, which reflects lytic cells.

Increased glycolytic flux, together with HIF1α stabilization, suggests that latent cells might be more adaptable to low oxygen conditions. We therefore tested whether miR-LEC proliferate faster than the control cells when subjected to such conditions. Cells were grown in high oxygen (20%) and then moved to 1.5% oxygen and analyzed after 24, 48 and 72 hours. In contrast to high oxygen conditions, where both control and miR-LEC growth rates were similar (Figure S6A), when cells were exposed to hypoxia, miR-LEC had a significant growth advantage in the first 24 hours (Figure 6A). This difference was less significant after 48 and 72 hours, suggesting that by these time points the control cells had adjusted to the low oxygen conditions. When we tested the effect of knocking down *EGLN2* and *HSPA9*, we found that only *EGLN2* silencing improved the initial response of LEC to hypoxia (Figure 6B and Figure S6B).

There is evidence that *in vitro* three-dimensional (3D) cell cultures more accurately reflect the complex *in vivo* microenvironment than simple two-dimensional cell monolayers. We therefore explored the effect of the KSHV miRNAs on cell growth under these conditions. To do this we used the recently published microplate-based method to create and measure spheroid growth [68]. To investigate the effect of the KSHV miRNAs in this context, we chose the human osteosarcoma U2OS cell line, which has previously been shown to form spheroids using this method. U2OS were infected with lentiviruses to express the miRNA cluster or the specific hairpins for *EGLN2* and *HSPA9* knockdown. As in LEC, expression of the KSHV miRNA cluster in U2OS led to up-regulation of HIF1α and down regulation of EGLN2 and HSPA9 (Figure S6C). These cells were then plated in ultra-low attachment 96-well round-bottomed plates as previously described [68]. Spheroid growth was measured using the CellTiter-Glo Luminescent Cell Viability Assay (Promega) and spheroid area was measured using the EVOS cell imaging system. We found that, similarly to the growth advantage under hypoxic conditions, expression of the KSHV miRNA cluster and knock down of EGLN2 led to enhanced growth in 3D cultures, as shown by increased spheroid area (Figure S6D–E) and cell number (Figure 6F).

The fact that KSHV has evolved to alter cellular metabolism and to reduce mitochondrial biogenesis during latency suggests that these phenomena have a function during the life cycle of the virus.

We therefore examined the importance of reduced mitochondrial biogenesis for latency maintenance. To test this, we treated the latently infected PEL cell line BCBL1 with Resveratrol, which has been shown to induce mitochondrial biogenesis in some cell types [69], [70]. We found that in PEL cells, Resveratrol treatment leads to increased expression of COX-IV and the OXPHOS complexes (Figure 6C–D), in a dose dependent manner. Strikingly, this increase in mitochondrial biogenesis caused extensive cell death (Figure 6E). When we tested for viral gene expression we found that Resveratrol treatment caused substantial expression of lytic genes (Figure 6F), suggesting that the Resveratrol-induced cell death is due to induction of the lytic cycle in these cells. In order to directly test for viral production we used the media from Resveratrol induced cells to directly infect 293T cells and measured the KSHV copy number in these cells. As shown in figure 6G, infection using media from cells treated with Resveratrol leads to increase of up to ∼15 times of KSHV copy number in the infected cells.

In order to determine if the same holds true in primary infected cells, we used the recombinant virus, rKSHV.219, which expresses a red fluorescent protein under the KSHV lytic PAN promoter [71]. This virus can establish a latent infection in LEC, making it a valuable model for the study of latency and reactivation. We selected infected LEC to create KLEC.219, as previously described [71], and treated them with Resveratrol to induce mitochondrial biogenesis. We first confirmed that Resveratrol does not have a toxic effect on LEC and that it induced mitochondrial biogenesis in them (Figure S7A and B). Resveratrol treatment led to extensive KSHV reactivation, as indicated by a 42% increase in RFP positive cells (Figure 6H and Figure S7C). Interestingly, when we overexpressed EGLN2 and HSPA9 in BCBCL1 and KLEC.219, we found that while HSPA9 did not show a significant effect, EGLN2 overexpression caused a small increase in lytic phase activation (Figure S7D and F). Respectively, knocking down HIF1α gave similar results; activation of the lytic phase indicated by increase in RFP positive cells and KSHV copy number in cell infected with media from these cells (Figure S7 E–F).

These results suggest that reduced mitochondrial activity is important for maintaining latency in KSHV infected cells.

## Discussion

KSHV has been shown to highjack and manipulate various cellular pathways to promote its own survival and spread [72]. Among those it was shown that KSHV alter its host cell energy metabolism, but neither the mechanism by which this is achieved nor the biological benefit, are understood. Our study has revealed a functional role for the KSHV miRNAs in the regulation of cell metabolism. We report that expression of the KSHV miRNA cluster, in primary LEC, induces the Warburg effect; it reduces oxygen consumption, increases lactate secretion, increases glucose uptake and reduces mitochondrial biogenesis. In addition, expression of these miRNAs also leads to stabilization and activation of the transcription factor HIF1α, a master regulator of cell metabolism.

We identified and confirmed two new targets for the KSHV miRNAs, EGLN2 and HSPA9, and show that KSHV alters host cell energy metabolism through down-regulation of these genes. Nevertheless, the fact that overexpression of these genes (without their 3′UTRs) did not fully rescue the phenotypic effect of these miRNAs, suggests that there are more genes involved in this metabolic transformation. This is not surprising because the miRNAs within the cluster are predicted to target many other genes, some of which are involved in cell metabolism, and these might also contribute to this phenomenon.

**Figure 7:**
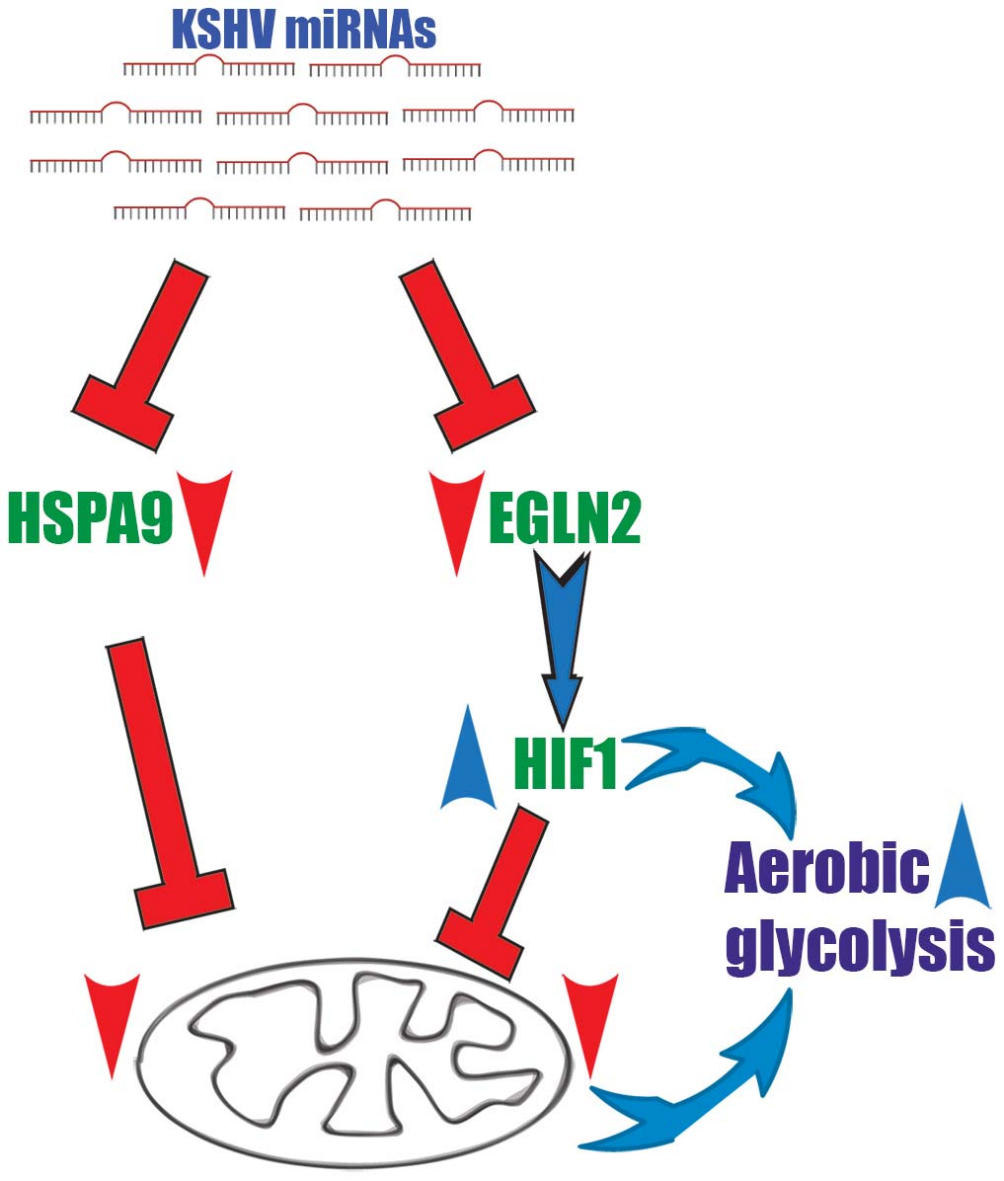
A two-armed mechanism model by which KSHV changes cellular energy metabolism. KSHV microRNAs induce a metabolic transformation by concurrent regulation of two independent pathways; transcriptional reprograming via HIF1 activation and reduction of mitochondria biogenesis through down regulation of the mitochondrial import machinery.

We suggest a two-armed mechanism by which KSHV changes cellular energy metabolism; the first is based on activation of the transcription factor HIF and the subsequent up-regulation of its metabolically relevant target genes. The second arm directly reduces mitochondrial biogenesis by impairing the import of proteins into mitochondria (Figure 7).

We show that the KSHV miRNAs induced-metabolic shift contributes to infected cells proliferation in low oxygen and is important for latency maintenance. KS and PEL cells are not exposed to extensive hypoxia and KSHV associated solid lymphomas are rare. Nevertheless, in some cases KS can have a solid mass [73], or PEL develops in hypoxic niches, as was shown in an artificial cavity related to the capsule of a breast implant [74]. In addition, adjustments to energy metabolism have been suggested to give cancer cells many advantages with respect to proliferation and growth. The prevalent theory is that, although aerobic glycolysis is an inefficient way to generate energy, it is a necessary adaption to facilitate the uptake and incorporation of nutrients into the biomass, which is needed to produce new cells. This modification also offers similar advantages for KSHV-infected cells.

We have shown here for the first time that the KSHV miRNAs regulate cell metabolism. Nevertheless, regulating energy and cancer metabolism using miRNAs is not exclusive to viruses, and cellular miRNAs are also known to control energy metabolism. As we show for the KSHV miRNAs, cellular miRNAs also participate in manipulating cancer cell metabolism by regulating the expression of genes with protein products that either directly regulate the metabolic machinery or indirectly modulate the expression of metabolic enzymes, serving as master regulators [75]–[78].

Various types of cancer cells have been shown to overexpress HIF due to intratumoral hypoxia or as a result of genetic alterations that have occurred as part of their oncogenic program (reviewed in [79]–[82]). We show a new miRNA-based mechanism by which KSHV regulates HIF1α levels in cells. The algorithm miRror [83] suggests that there are 35 cellular miRNAs predicted to target *EGLN2* (table S2) and 14 (table S3) predicted to target all three HIF prolyl hydroxylases. This suggests that a similar miRNA-based regulation mechanism of HIF1α stability might exist in normal or cancerous cells.

All previously performed AGO2 CLIP experiments in PEL, several different prediction algorithms and our results suggest that the KSHV miRNAs target *HSPA9*, together with other proteins from the mitochondrial import machinery. We confirm that several of the KSHV miRNAs collaborate to target *HSPA9* and consequently reduce mitochondrial activity. Very little is known about how cells regulate import into mitochondria and whether this might be a mechanism through which mitochondrial biogenesis and activity is regulated. Saccharomyces cerevisiae, which do not have miRNAs, regulate import into mitochondria using cytosolic kinases [84]. We propose a possible regulation of the mitochondrial import machinery by miRNAs. Our results suggest the KSHV miRNAs regulates key proteins in this import machinery such as HSPA9, TOMM40, TOMM22 and others. This could be the reason that over expression of EGLN2 and HSPA9 failed to rescue the miRNAs phenotype, since other proteins from the import machinery are still down regulated. Regulation of this process by miRNAs could allow fine-tuning of mitochondrial activity, which could be rapidly reversed. It has been previously shown that knockout of the mitochondrial HSP70 (HSPA9), as well as other essential proteins in the import machinery, is lethal to cells. Thus, fine-tuning achieved by miRNAs can allow viruses to reduce mitochondrial oxidative phosphorylation while keeping its host cell alive.

Our findings provide further support, in the context of cell metabolism, for the concept that KSHV miRNAs operate as a functional cluster. We show that KSHV miRNAs are expressed together in infected cells and collaborate to regulate specific genes (e.g. EGLN2 and HSPA9) within specific pathways (e.g. the mitochondria import machinery). With regards to individual mRNA targets we observe synergy but also some redundant targeting. However, we find that no individual miRNA can fully reproduce the effect of the whole KSHV miRNA cluster on metabolism, suggesting that these miRNAs function synergistically. Moreover, the fact that they are not all expressed to same level suggests an internal regulation of their expression. We speculate that by expressing this cluster, the virus circumvents one of the host cell's main evolutionary tools for escaping unwanted miRNA targeting: alterations in 3′UTR sequences. Even if one miRNA target site is lost, the remaining miRNAs are still able to bind to and regulate the transcript. Although the specific contribution of each of the KSHV miRNAs to this phenomenon has not yet been dissected, through suppressing expression of multiple genes within a specific cellular pathway, the overall effect of the miRNA cluster is coherent, robust and quantitatively sufficient to lead to functionally relevant outcomes. Interestingly, this functional clustering of viral miRNAs around cellular metabolism seems to be conserved in other herpesviruses. EBV, also a gamma-herpesvirus, which expresses multiple viral miRNAs, regulates many of the same genes and cellular pathways as KSHV. Notably, an AGO2 CLIP experiment [16] pulled HSPA9, together with other proteins involved in import into mitochondria, as targets of the EBV miRNAs, suggesting potential functional convergence.

KSHV-infected cells share many characteristics with cancer cells, in particular altered energy metabolism. Therefore, in addition to the implications for KS biology, KSHV infection could be used as a model to study the role of the Warburg effect (and other altered metabolic pathways) during transformation.

Although having a growth advantage in hypoxic conditions may be significant in certain environments, we speculate that the main evolutionary benefit gained by KSHV through alteration of cellular metabolism is the generation of optimal intracellular conditions for latency establishment and maintenance. Our results show that inducing mitochondria biogenesis interferes with the virus ability to maintain latency - a key step in the virus oncogenic program. Consequently these findings have translational-medicine implications with regards to KSHV-associated malignancies, but also in the broader context of pathologies etiologically linked to DNA viruses.

## Materials and Methods

### Cell culture

LEC were purchased from Promocell and grown in endothelial growth medium MV2 (Promocell). LEC were used for experiments before passage 8. BCLB-1 cells latently infected with recombinant GFP-KSHV [47] were cultured in RPMI 1640 (Invitrogen) supplemented with 10% FCS and 400 ng/mL Geneticin (Invitrogen). 293T, U2OS and Vero cells were grown in DMEM (Invitrogen), supplemented with 10% FBS.

### Plasmids

The KSHV miRNA cluster and individual miRNAs were cloned into the lentiviral vector pSIN-MCS as previously described [18]. The cluster and individual miRNAs were subcloned into the gateway entry vector pENTR/pTER+ [85]. These miRNAs were further cloned into the 3rd gen lentiviral promoter-less Gateway destination vectors pLenti X1 Puro DEST and pLenti CMV GFP DEST using the Gateway LR Clonase II enzyme mix (Invivogen). HSPA9 was amplified using specific primers (Table S4) and cloned into pENTR4. The gene was further cloned into pLenti PGK Puro Dest [85] using the Gateway LR Clonase II enzyme mix (Invitrogen). FLAG-EglN2-pLenti6 was as previously described [85].

### Lentivirus production and infection of LEC

Vesicular stomatitis virus-G envelope-pseudotyped lentiviral virions were produced by cotransfecting 6 µg lentiviral construct, 3 µg p8.91, and 1 µg pMD.G into a 10-cm dish of ∼70% confluent 293T cells using the FuGENE (Roche) protocol. Five hours after transfection, the medium was changed, and 48 h after transfection, the medium containing the lentiviral virions was collected, passed through a 0.45 µm filter, and either aliquoted directly or concentrated and stored at −80°C. Lentiviral infections were done by incubating the desired amount of virus preparation with suspension LEC for 5 h, after which the medium was changed.

Titration of each lentivirus preparation was done either by quantitative PCR [86] or Flow cytometry [87]. All experiments were performed in infected LEC showing more than 80% positive cells.

### Basal cellular respiration rate

Cells were seeded in XF 24-well cell culture microplates (Seahorse Bioscience) at 4×10^4^ cells/well (0.32 cm^2^) in 200 µl growth medium and then incubated at 37°C/5% CO_2_ for 20–24 hours. Assays were initiated by removing the growth medium from each well and replacing it with 600 µl of assay medium pre-warmed to 37°C. The cells were incubated at 37°C for 30 minutes to allow media temperature and pH to reach equilibrium before the first rate measurement. Prior to each rate measurement, the XF24 Analyzer gently mixed the assay media in each well for 3 min to allow the oxygen partial pressure to reach equilibrium. Following mixing, OCR and ECAR were measured simultaneously for 4 min to establish a baseline rate. The assay medium was then gently mixed again for 3 min between each rate measurement to restore normal oxygen tension and pH in the microenvironment surrounding the cells. Uncoupled, maximal and non-mitochondrial respiration was determined after the addition of 5 µM oligomycin, 1 µM carbonyl cyanide 4-(trifluoromethoxy)phenylhydrazone (FCCP) and 2 µM antimycin-A. All chemicals were from Sigma-Aldrich.

### Western blotting and antibodies

Cells were lysed in RIPA buffer (300 mM Sodium Chloride, 1% NP-40, 0.5% Sodium deoxycholate, 0.1% Sodium dodecyl sulphate and 50 mM Tris pH 8.0). Equal amounts of protein were resolved on Mini-PROTEAN TGX Precast gels (Bio-Rad Laboratories). Antibodies against EGLN2 (Novus Biologicals), HSPA9 (Abgent), GLUT1 (Alpha Diagnostic), HSP90 (Cell signaling), α-tubulin and Flag (Sigma-Aldrich) were detected with IRDye secondary antibodies (LI-COR). Antibodies against HIF1α (BD Transduction Laboratories) and the MitoProfile Total OXPHOS Human WB Antibody Cocktail (Abcam) were detected with HRP-conjugated secondary antibodies and were quantified using ECL (GE Healthcare). Images were analysed using the Image Studio Lite (LI-COR) or the ImageQuant (GE) Western blot analysis softwares.

### qPCR and qRT–PCR

Genomic DNA for qPCR was extracted using the QIAamp DNA mini-kit (Qiagen). Total RNA was extracted using either the RNeasy mini-kit or the miRNeasy mini-kit (Qiagen). Approximately 100 to 1000 ng of total RNA was used for cDNA synthesis using the ProtoScript II Reverse Transcriptase (New England Biolabs). DNA and mRNA levels were quantified by qPCR and qRT-PCR using optimized primers (Table S4) and SYBR Green PCR master mix (Applied Biosystems). qRT-PCR quantification of *VEGF* and ADM was performed using Taqman gene expression assays (Applied Biosystems).

cDNA synthesis for qRT–PCR quantification of mature miRNAs was performed using the Exiqon Universal cDNA Synthesis Kit II according to the manufacturer's instructions. Detection of the mature KSHV miRNAs was performed using the kshv-miR LNA PCR primer sets (Exiqon). Cellular small nucleolar RNA *RNU66* was used as a reference RNA.

### Cell count and viability

Cells were counted and analyzed for viability using the Muse Count & Viability Reagent on the Muse Cell Analyzer (Merck Millipore).

### Glucose uptake

Glucose uptake was measured by incubating cells with 30 µM glucose analogue 6-NBDG (Invitrogen) for 15 minutes. Cells where then washed and trypsinized and their fluorescence (λex: 465 nm, λem: 540 nm) was measured by flow cytometry.

### 3′UTR luciferase reporter assay

The 3′UTRs of the indicated genes were amplified using specific primers (Table S4) and cloned into the psiCHECK 2 vector (Promega). The reporter plasmids (50 ng) were transfected into 293T cells, 48 hours after transfection with the individual miRNAs or the miRNA cluster. Cells were harvested 24 hours after transfection according to the Dual-Luciferase Reporter assay system (Promega). Luciferase activity was measured using a Fluoroskan Ascent FL luminometer (ThermoScientific). Firefly activity was normalized to internal Renilla luciferase levels.

### HRE luciferase reporter assay

U2OS-HRE-luc cells were infected with the control or the miRNA cluster vector. 72 hours post infection cells were washed twice with ice-cold PBS and lysed using Glo Lysis buffer (Promega). Luciferase activity was measured using the steady Glo Luciferase assay (Promega).

### Mitochondrial structure and volume

Cells were loaded with 5 µM Calcein-AM and 50 nM MitoTracker Red (Invitrogen; 37°C, 30 minutes) in growth media for 30 minutes and Z-series of images were acquired using a Zeiss LSM 510 system (Carl Zeiss, Inc., Cambridge, UK), as previously described [88]. Maximal projection of images was used to quantify the area of green (Calcein) and red (mitoTracker Red) signal. Mitochondrial area was defined relative to cytoplasmic area as ‘area red/area green’. Images were analyzed using the MetaMorph Microscopy Automation & Image Analysis Software (Molecular Devices). The two channels (Calcein-AM and MitoTracker Red) were separated and threshold in order to acquire two separate binary images.

### KSHV preparation and establishment of stable infections

rKSHV.219 stocks were prepared from the Vero cell as previously described [71]. Stably infected LEC were selected and maintained with 1 ug/ml puromycin (Invivogen).

### SRB assay

Cells were grown in 96well plates and fixed using cold 10% Trichloroacetic acid for 1 hour at 4°C. Wells were then washed 5 times with distilled water and left to air dry before staining with of 0.4% w/v Sulforhodamine B (Sigma-Aldrich) for 30 minutes at room temperature. Wells were washed 5 times with 1% v/v acetic acid and left to air dry before adding 100 µl of 10 mM Tris base pH 10.5. Plates were incubated on a plate shaker for 5 minutes and read at 564 nm using the Varioskan Flash plate reader (Thermo Scientific).

### Generation and analysis of spheroids

For spheroid generation, 200 µl of cell suspension (1×10^4^ cells/ml) were dispensed into ULA 96-well round-bottomed plates (Corning) using a multichannel pipette. Plates were incubated for 4 days at 37°C, 5%CO_2_. Images were acquired by EVOS cell imaging system and analysed using the Adobe Photoshop CS6 extended version.

## Supporting Information

Figure S1 **KSHV miRNA cluster induces aerobic glycolysis and stabilizes HIF 1 alpha.** LEC, infected with lentivirus expressing LANA, vcyclin, vFLIP or the miRNA cluster, were harvested 72 hours post-infection. **A–D.** Relative mRNA levels (ΔCt) for *LANA*, *vcyclin* and *vFLIP* were determined by quantitative real-time PCR (qRT-PCR). Expression was measured relative to the *TUBB*. **E–F.** cDNA synthesis was performed using the Exiqon Universal cDNA Synthesis Kit II. Detection of the mature KSHV miRNAs was performed using the KSHV-miR LNA PCR primer sets (Exiqon). Expression was measured relative to the cellular small nucleolar RNA RNU66. **G.** OCR of cells expressing the different components of the oncogenic cluster was measured using the Seahorse XF24 Analyzer. Cells were seeded at density of 4×10^4^ cells per well and the assay was performed according to the manufacturer's protocol. Uncoupled, maximal and non-mitochondrial respiration was determined after the addition of 5 µM oligomycin, 1 µM carbonyl cyanide 4-(trifluoromethoxy)phenylhydrazone (FCCP) and 2 µM antimycin-A. **H.** Base line OCR was measured using the Seahorse XF24 Analyser in Control, miR-LEC and miR-LEC expressing also miR-K10-12 and miR-K12-12. **I.** Lactate levels in the control and miR-LEC culture media. Equal numbers of cells were grown for 24 hours and lactate levels in the media were measured using the MBL Lactate Colorimetric assay kit. The bar graph presents the average ratio between control and miR-LEC from 3 independent experiments (Mean+SEM, n = 3). **J–L**. Glucose uptake into: LEC expressing the different components of the oncogenic cluster (J), miR-LEC and miR-LEC expressing also miR-K12-10 and miR-K12-12 (K) and LEC expressing the miR-132/212 cluster (L). Cells were incubated with 30 µM of the fluorescent glucose analogue 6-NBDG for 20 minutes prior to analysis by fluorescence-activated cell sorter (FACS). **M.** Base line OCR was measured using the Seahorse XF24 Analyser in Control, LEC expressing the miR-132/212 cluster and miR-LEC. **N.** U2OS-HRE-luc cells were infected with the control or the miRNA cluster vectors. 72 hours post infection cells were washed twice with ice-cold PBS and lysed using Glo Lysis buffer (Promega). Luciferase activity was measured using the steady Glo Luciferase assay (Promega) according to the manufacturer's instructions.
(TIF)

Figure S2 **Expression of the KSHV miRNA cluster reduces mitochondrial biogenesis.** **A–B.** Mitochondrial volume in miR-LEC. Cells were loaded with 5 µM Calcein-AM and 5 nM MitoTracker Deep Red FM and Z-series of images were. Maximal projections of images were used to quantify the area of green (Calcein) and red (MitoTracker Deep Red) signals as previously described [88]. The bar graph on the right presents the average relative mitochondrial volume in miR-LEC compared to control cells (Mean±SEM, n = 3). **C.** Expression levels of the 5 OXPHOS complexes as measured by Western blotting analysis using the MitoProfile Total OXPHOS Human WB Antibody Cocktail in LEC expressing either a control vector or the viral miRNA cluster. The left and right panels are two exposures of the same Western blot presented in figure 2. **D.** Relative values of different MitoProfile antibodies. The graph presents the total intensities of the different detected antibodies normalized to HSP90 (Mean+SEM, n = 3).
(TIF)

Figure S3 **The KSHV miRNA cluster regulates EGLN2 and HSPA9.** **A.** Expression levels of *EGLN1*, *EGLN2* and *EGLN3* in primary LEC. mRNA levels were determined by qRT-PCR. Tubulin beta (*TUBB*) levels were used for normalization. **B.** Relative mRNA levels of *EGLN1*, *EGLN2* and *EGLN3* in miR-LEC compared to control cells. mRNA levels were determined by qRT-PCR. *TUBB* levels were used for normalization. **C.** Relative mRNA levels of 9 genes from the mitochondrial import machinery in miR-LEC compared to control cells. mRNA levels were determined by qRT-PCR. *TUBB* levels were used for normalization. **D.** Reporter assay indicating the response of HSPD1, TOMM22, TOMM40 and TIMM23 3′UTRs to the KSHV miRNA cluster. Firefly expression was normalized to Renilla expression to give the relative light units (RLU), which are shown relative to the non-targeting control. **E.** Relative protein expression for EGLN2 and HSPA9 from 3 independent experiments were calculated according to the signal measured using the Odyssey (Mean+SEM, n = 3). **F.** Reporter assay indicating the sensitivity of the *EGLN2* or *HSPA9* 3′UTRs to targeting by the KSHV miRNA cluster (Mean+SEM, n = 3). Firefly expression was normalized to Renilla expression to give the relative light units (RLU), which are shown relative to the non-targeting control. In all panels statistical significance denoted by *P<.05; **P<.01; ***P<.001. **G.** Relative mRNA levels of *EGLN2* and *HSPA9* in KLEC compared to control cells. mRNA levels were determined by qRT-PCR. TUBB levels were used for normalization. **H.** Expression of the mature KSHV miRNAs when expressed in LEC individually. Detection of the mature KSHV miRNAs was performed using the KSHV-miR LNA PCR primer sets (Exiqon).
(TIF)

Figure S4 **Expression levels of EGLN2, HSPA9 and the HIF1 alpha P402A/P564A stable mutant.** **A–B.** Relative mRNA levels of *EGLN2* (A) and *HSPA9* (B) in LEC infected with specific hairpins for *EGLN2* and *HSPA9* (Open Biosystems). mRNA levels were determined by qRT-PCR. TUBB levels were used for normalization. **C.** Relative protein expression for EGLN2 and HSPA9 from 3 independent experiments were calculated according to the signal measured using the Odyssey (Mean+SEM, n = 3). **D.** HIF1 alpha protein expression, as measured by Western blotting, in LEC infected with lentivirus expressing the HIF1 alpha P402A/P564A stable mutant.
(TIF)

Figure S5 **Overexpression of EGLN2 and HSPA9 partially rescues the miRNA cluster effect on glucose metabolism.** **A.** Protein expression, as measured by Western blotting using anti Flag antibody, in miR-LEC infected with lentivirus expressing either EGLN2-Flag or HSPA9-Flag. **B.** Relative mRNA levels of *EGLN2* and *HSPA9* in miR-LEC expressing EGLN2-Flag or HSPA9-Flag. mRNA levels were determined by qRT-PCR. *TUBB* levels were used for normalization.
(TIF)

Figure S6 **The miRNAs induced metabolic shift enhanced growth under hypoxia and in 3D culture.** **A–B.** 7500 cells expressing the KSHV miRNA cluster or non-targeting control (A) and cells expressing the shControl or shEGLN2 (B) were plated in 96 well plates. Cells were fixed after 30 minutes, 24, 48 and 72 hours using 10% Trichloroacetic acid, stained with Sulforhodamine B, and then plates were read at 564 nm. Optical density indicates the amount of proteins in the different wells. **C.** Protein expression, as measured by Western blotting, in selected U2OS cells expressing the KSHV miRNA cluster. **D–F.** 5000 cells of each condition were plated in ultra-low attachment 96-well round-bottomed plates. Spheroids were imaged at day 5,9 and 14 and analyzed using Adobe Photoshop CS6 for spheroid area (D–E), or harvested using CellTiter-Glo Luminescent Cell Viability Assay (F).
(TIF)

Figure S7 **The miRNAs induced metabolic shift is important for latency maintenance.** **A.** LEC were treated with the indicated concentration of Resveratrol for 48 hours. Viability was determined using the Muse Count & Viability Assay Kit on the Muse cell analyzer (Merck Millipore). **B.** Mitochondrial DNA (mtDNA) copy number in cells treated with the indicated Resveratrol concentrations. qPCR was carried out as described in [89]. **C–E.** Flow cytometry analysis of KLEC.219 (GFP positive) for RFP expression. Cells were treated with Resveratrol or TPA at the indicated dose and time period, or infected with the indicated lentiviruses. The numbers denote the percentage of RFP positive cells, which reflects lytic cells. **F.** Relative KSHV DNA copy number in 293T cells infected using the growth media of BCBL1 cell infected with the indicated lentiviruses.
(TIF)

Table S1 **KSHV miRNAs that are predicted by the PITA algorithm to target EGLN2 or HSPA9 and found to down-regulate the mRNA levels of these genes or to reduce luciferase activity in 3′UTR assay (shown in** **Figure 3C and D** **).**
(PDF)

Table S2 **Cellular miRNAs which are predicted to target EGLN2 according to the algorithm miRror [83]** **.**
(PDF)

Table S3 **Cellular miRNAs which are predicted to target all three HIF prolyl hydroxylase (EGLN1-3) according to the algorithm miRror [83]** **.**
(PDF)

Table S4 **List of primers used in this study.**
(PDF)
