# Supplementary figures and images for: Kaposi's Sarcoma Herpesvirus MicroRNAs Induce Metabolic Transformation of Infected Cells
Source: PLoS Pathog. 2014 Sep 25;10(9):e1004400. doi: 10.1371/journal.ppat.1004400 (PMC4177984; doi:10.1371/journal.ppat.1004400)

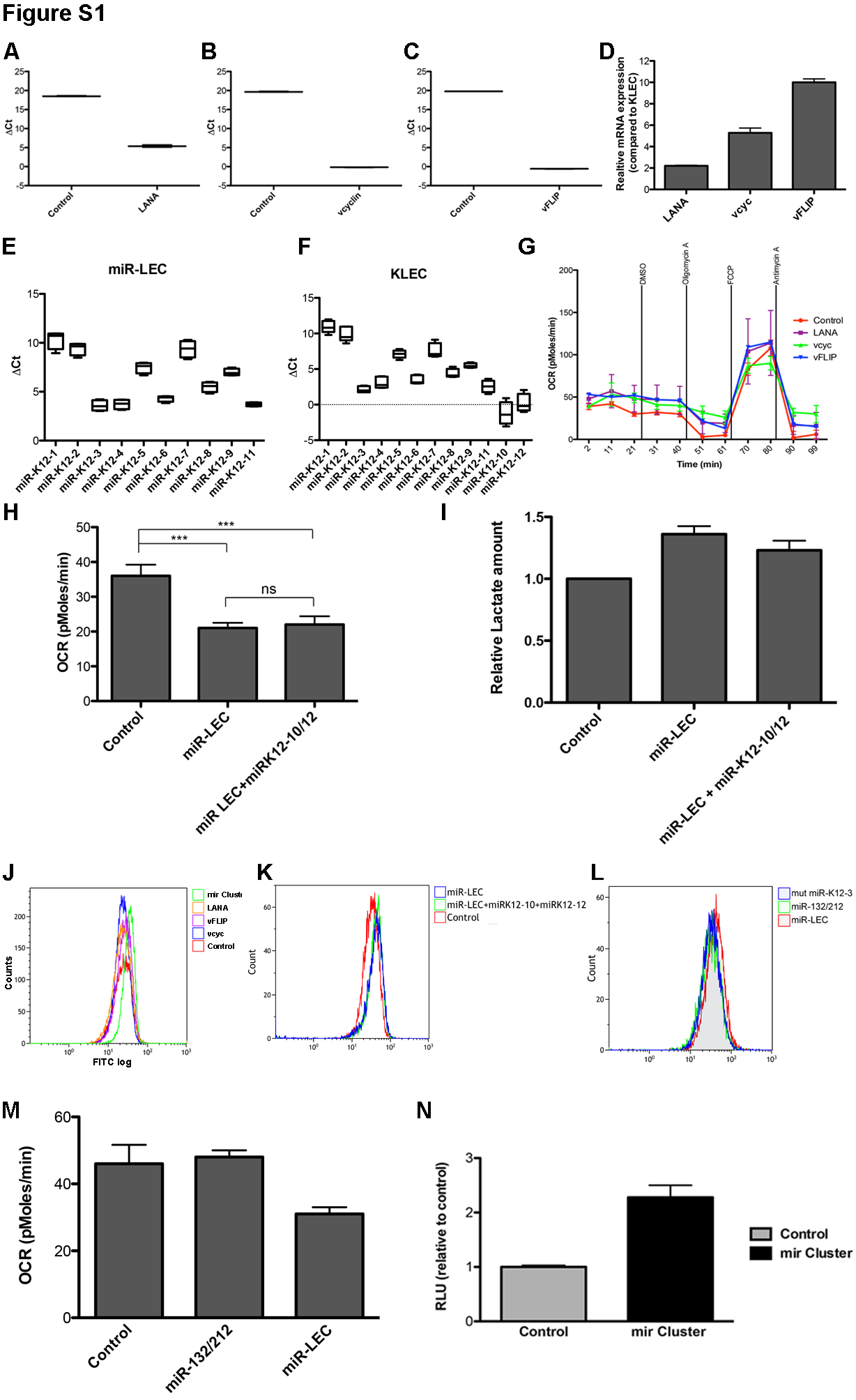

Supplement: Figure S1 — KSHV miRNA cluster induces aerobic glycolysis and stabilizes HIF 1 alpha. LEC, infected with lentivirus expressing LANA, vcyclin, vFLIP or the miRNA cluster, were harvested 72 hours post-infection. A–D. Relative mRNA levels (ΔCt) for LANA, vcyclin and vFLIP were determined by quantitative real-time PCR (qRT-PCR). Expression was measured relative to the TUBB. E–F. cDNA synthesis was performed using the Exiqon Universal cDNA Synthesis Kit II. Detection of the mature KSHV miRNAs was performed using the KSHV-miR LNA PCR primer sets (Exiqon). Expression was measured relative to the cellular small nucleolar RNA RNU66. G. OCR of cells expressing the different components of the oncogenic cluster was measured using the Seahorse XF24 Analyzer. Cells were seeded at density of 4×10^4^ cells per well and the assay was performed according to the manufacturer's protocol. Uncoupled, maximal and non-mitochondrial respiration was determined after the addition of 5 µM oligomycin, 1 µM carbonyl cyanide 4-(trifluoromethoxy)phenylhydrazone (FCCP) and 2 µM antimycin-A. H. Base line OCR was measured using the Seahorse XF24 Analyser in Control, miR-LEC and miR-LEC expressing also miR-K10-12 and miR-K12-12. I. Lactate levels in the control and miR-LEC culture media. Equal numbers of cells were grown for 24 hours and lactate levels in the media were measured using the MBL Lactate Colorimetric assay kit. The bar graph presents the average ratio between control and miR-LEC from 3 independent experiments (Mean+SEM, n = 3). J–L. Glucose uptake into: LEC expressing the different components of the oncogenic cluster (J), miR-LEC and miR-LEC expressing also miR-K12-10 and miR-K12-12 (K) and LEC expressing the miR-132/212 cluster (L). Cells were incubated with 30 µM of the fluorescent glucose analogue 6-NBDG for 20 minutes prior to analysis by fluorescence-activated cell sorter (FACS). M. Base line OCR was measured using the Seahorse XF24 Analyser in Control, LEC expressing the miR-132/212 cluster and m [file ppat.1004400.s001.tif]

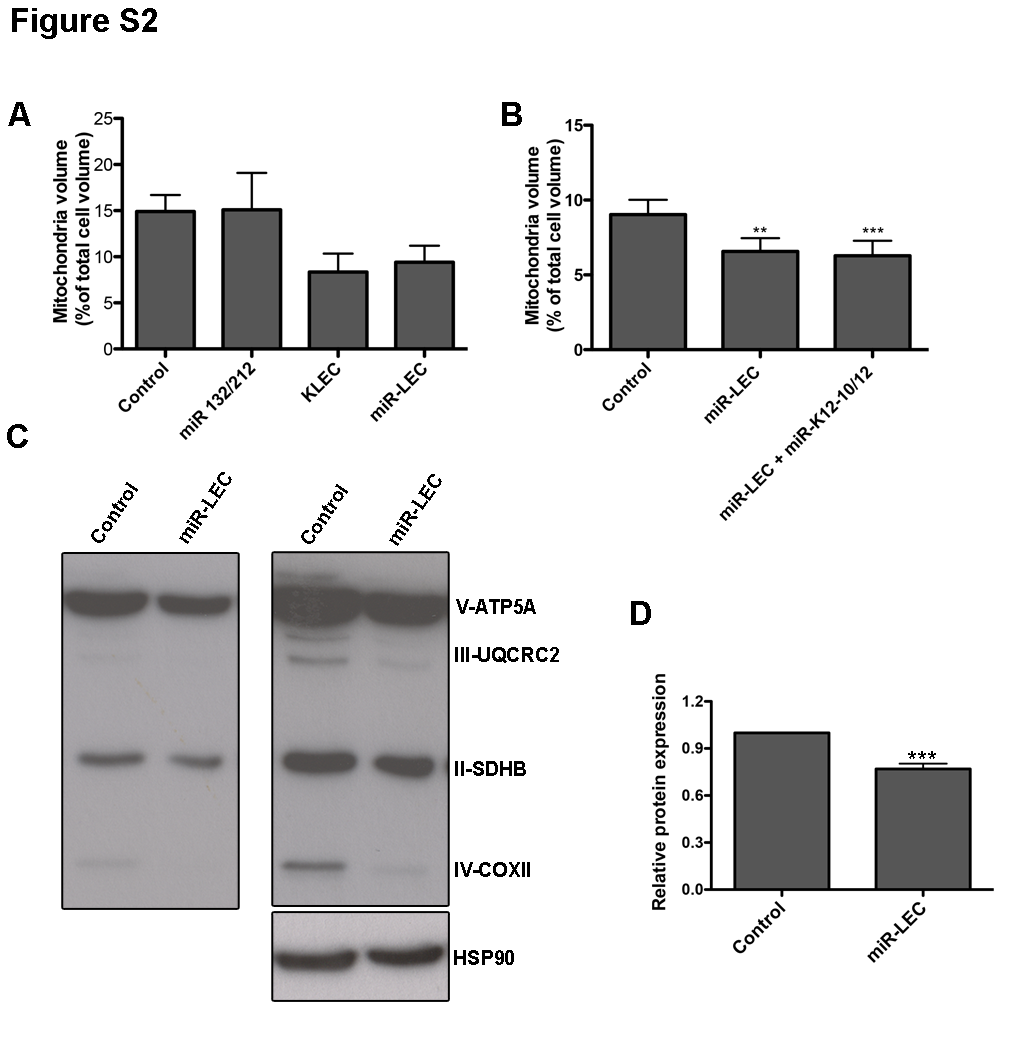

Supplement: Figure S2 — Expression of the KSHV miRNA cluster reduces mitochondrial biogenesis. A–B. Mitochondrial volume in miR-LEC. Cells were loaded with 5 µM Calcein-AM and 5 nM MitoTracker Deep Red FM and Z-series of images were. Maximal projections of images were used to quantify the area of green (Calcein) and red (MitoTracker Deep Red) signals as previously described [88]. The bar graph on the right presents the average relative mitochondrial volume in miR-LEC compared to control cells (Mean±SEM, n = 3). C. Expression levels of the 5 OXPHOS complexes as measured by Western blotting analysis using the MitoProfile Total OXPHOS Human WB Antibody Cocktail in LEC expressing either a control vector or the viral miRNA cluster. The left and right panels are two exposures of the same Western blot presented in figure 2. D. Relative values of different MitoProfile antibodies. The graph presents the total intensities of the different detected antibodies normalized to HSP90 (Mean+SEM, n = 3). (TIF) [file ppat.1004400.s002.tif]

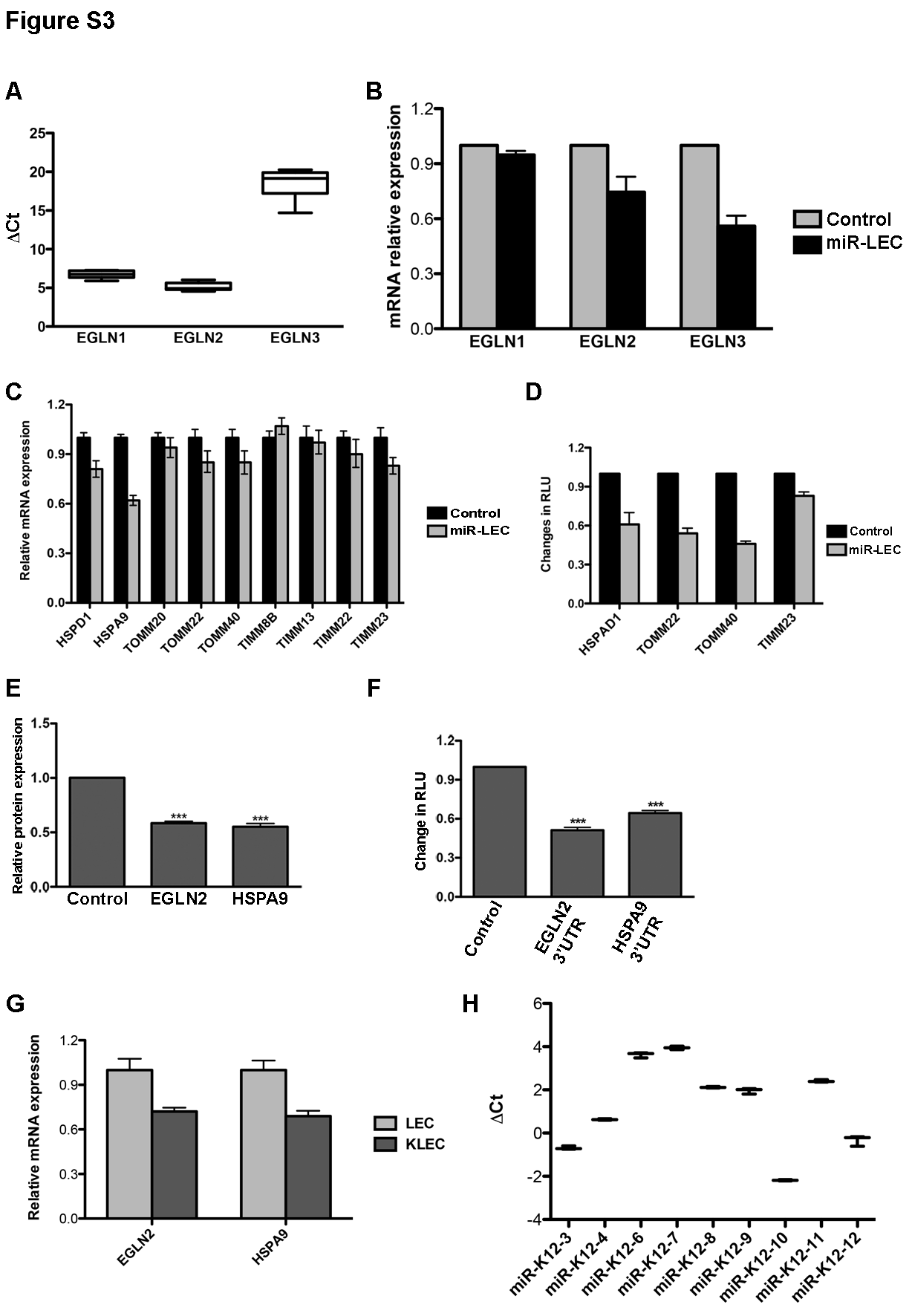

Supplement: Figure S3 — The KSHV miRNA cluster regulates EGLN2 and HSPA9. A. Expression levels of EGLN1, EGLN2 and EGLN3 in primary LEC. mRNA levels were determined by qRT-PCR. Tubulin beta (TUBB) levels were used for normalization. B. Relative mRNA levels of EGLN1, EGLN2 and EGLN3 in miR-LEC compared to control cells. mRNA levels were determined by qRT-PCR. TUBB levels were used for normalization. C. Relative mRNA levels of 9 genes from the mitochondrial import machinery in miR-LEC compared to control cells. mRNA levels were determined by qRT-PCR. TUBB levels were used for normalization. D. Reporter assay indicating the response of HSPD1, TOMM22, TOMM40 and TIMM23 3′UTRs to the KSHV miRNA cluster. Firefly expression was normalized to Renilla expression to give the relative light units (RLU), which are shown relative to the non-targeting control. E. Relative protein expression for EGLN2 and HSPA9 from 3 independent experiments were calculated according to the signal measured using the Odyssey (Mean+SEM, n = 3). F. Reporter assay indicating the sensitivity of the EGLN2 or HSPA9 3′UTRs to targeting by the KSHV miRNA cluster (Mean+SEM, n = 3). Firefly expression was normalized to Renilla expression to give the relative light units (RLU), which are shown relative to the non-targeting control. In all panels statistical significance denoted by *P<.05; **P<.01; ***P<.001. G. Relative mRNA levels of EGLN2 and HSPA9 in KLEC compared to control cells. mRNA levels were determined by qRT-PCR. TUBB levels were used for normalization. H. Expression of the mature KSHV miRNAs when expressed in LEC individually. Detection of the mature KSHV miRNAs was performed using the KSHV-miR LNA PCR primer sets (Exiqon). (TIF) [file ppat.1004400.s003.tif]

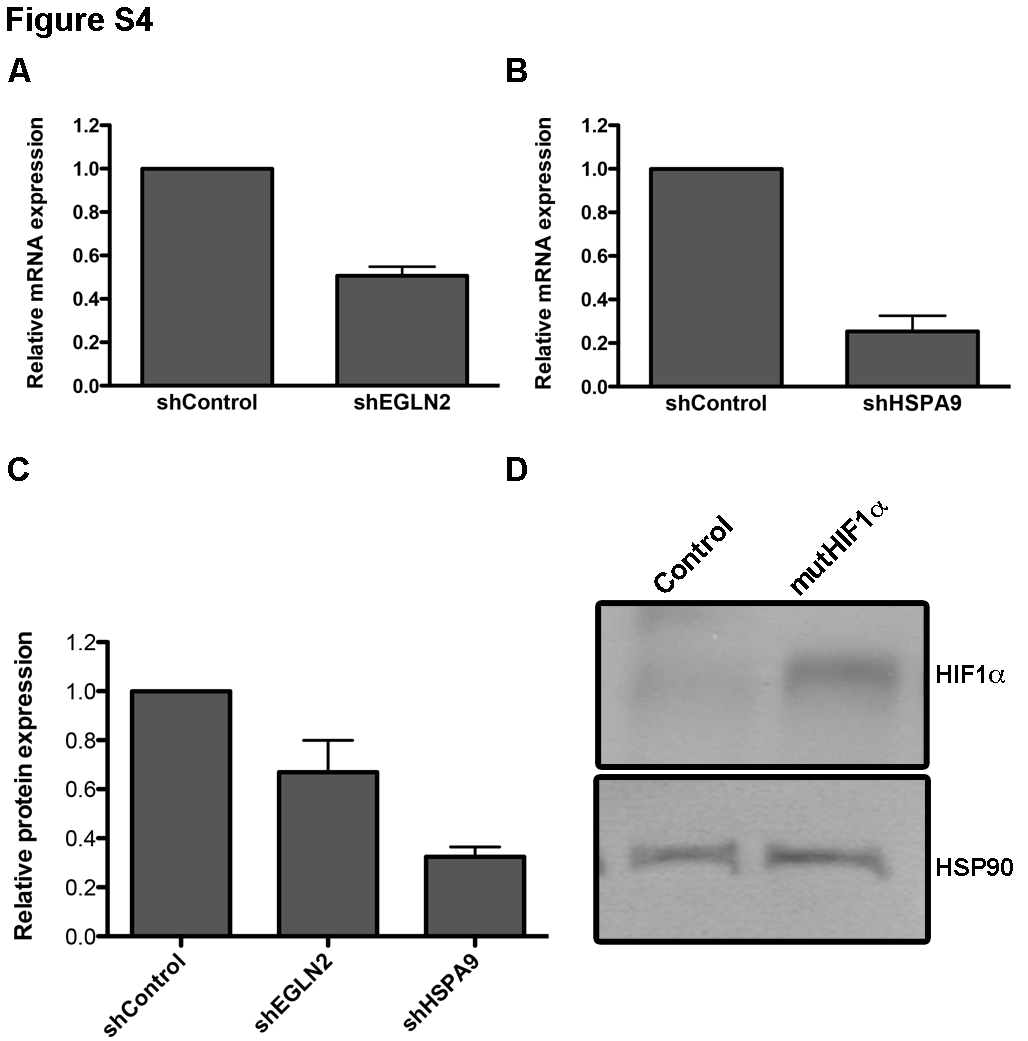

Supplement: Figure S4 — Expression levels of EGLN2, HSPA9 and the HIF1 alpha P402A/P564A stable mutant. A–B. Relative mRNA levels of EGLN2 (A) and HSPA9 (B) in LEC infected with specific hairpins for EGLN2 and HSPA9 (Open Biosystems). mRNA levels were determined by qRT-PCR. TUBB levels were used for normalization. C. Relative protein expression for EGLN2 and HSPA9 from 3 independent experiments were calculated according to the signal measured using the Odyssey (Mean+SEM, n = 3). D. HIF1 alpha protein expression, as measured by Western blotting, in LEC infected with lentivirus expressing the HIF1 alpha P402A/P564A stable mutant. (TIF) [file ppat.1004400.s004.tif]

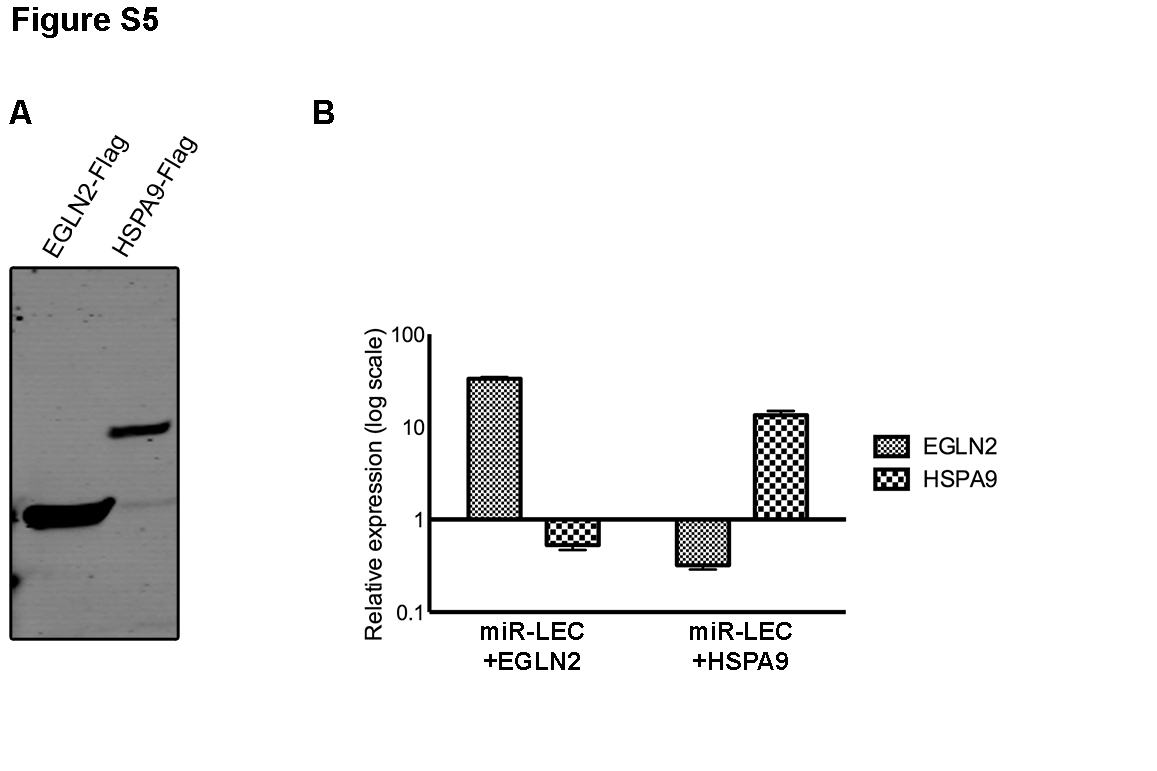

Supplement: Figure S5 — Overexpression of EGLN2 and HSPA9 partially rescues the miRNA cluster effect on glucose metabolism. A. Protein expression, as measured by Western blotting using anti Flag antibody, in miR-LEC infected with lentivirus expressing either EGLN2-Flag or HSPA9-Flag. B. Relative mRNA levels of EGLN2 and HSPA9 in miR-LEC expressing EGLN2-Flag or HSPA9-Flag. mRNA levels were determined by qRT-PCR. TUBB levels were used for normalization. (TIF) [file ppat.1004400.s005.tif]

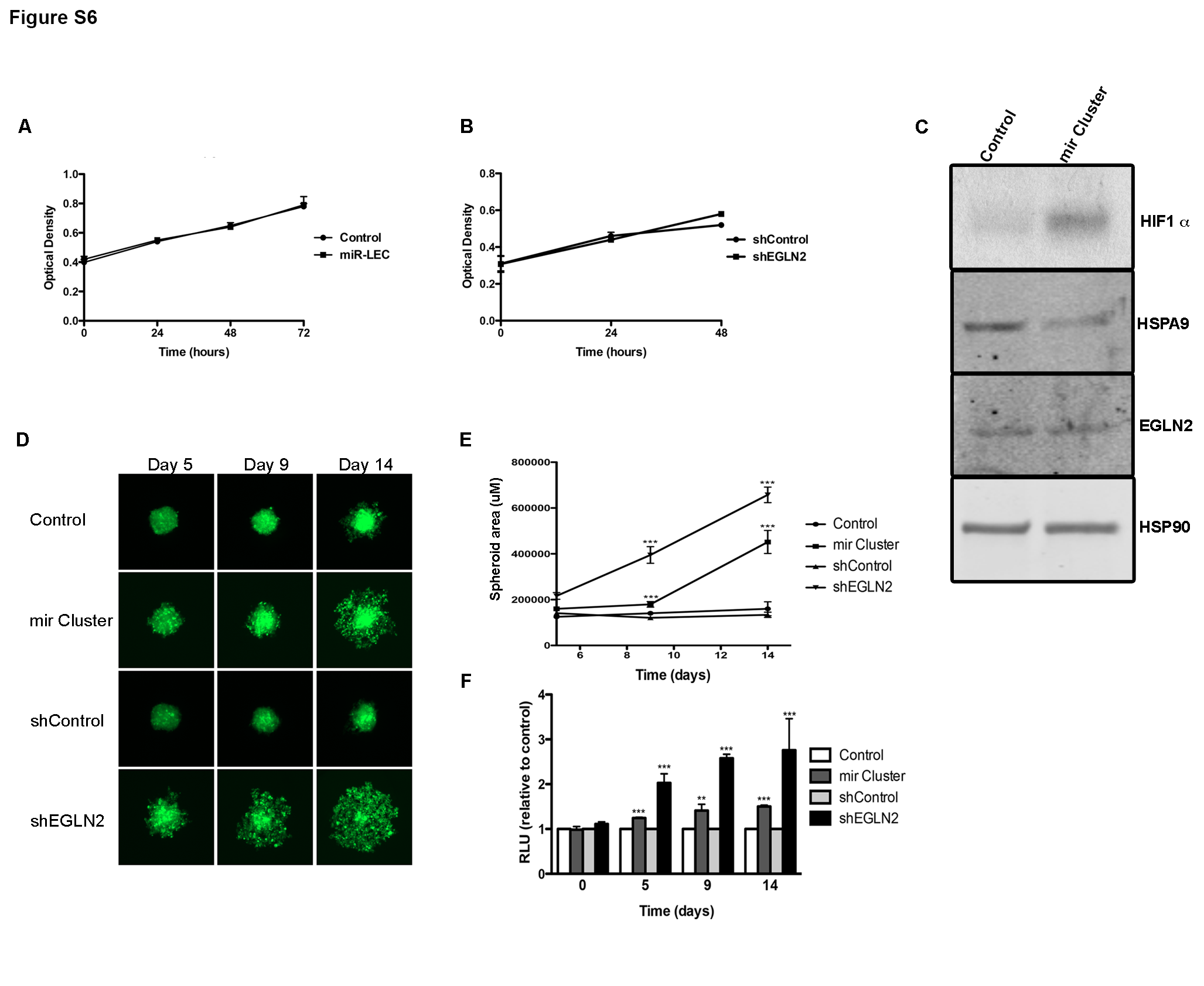

Supplement: Figure S6 — The miRNAs induced metabolic shift enhanced growth under hypoxia and in 3D culture. A–B. 7500 cells expressing the KSHV miRNA cluster or non-targeting control (A) and cells expressing the shControl or shEGLN2 (B) were plated in 96 well plates. Cells were fixed after 30 minutes, 24, 48 and 72 hours using 10% Trichloroacetic acid, stained with Sulforhodamine B, and then plates were read at 564 nm. Optical density indicates the amount of proteins in the different wells. C. Protein expression, as measured by Western blotting, in selected U2OS cells expressing the KSHV miRNA cluster. D–F. 5000 cells of each condition were plated in ultra-low attachment 96-well round-bottomed plates. Spheroids were imaged at day 5,9 and 14 and analyzed using Adobe Photoshop CS6 for spheroid area (D–E), or harvested using CellTiter-Glo Luminescent Cell Viability Assay (F). (TIF) [file ppat.1004400.s006.tif]

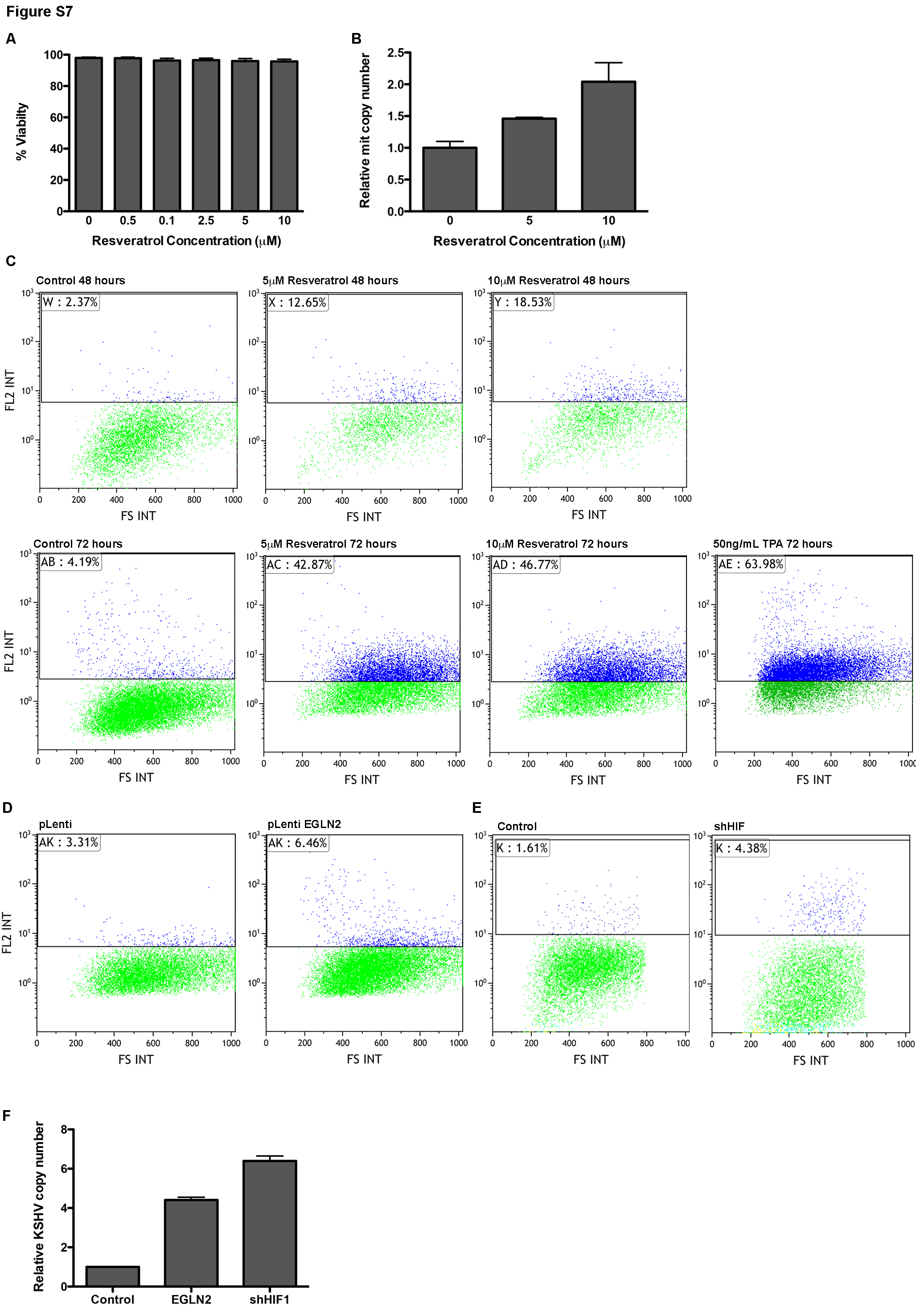

Supplement: Figure S7 — The miRNAs induced metabolic shift is important for latency maintenance. A. LEC were treated with the indicated concentration of Resveratrol for 48 hours. Viability was determined using the Muse Count & Viability Assay Kit on the Muse cell analyzer (Merck Millipore). B. Mitochondrial DNA (mtDNA) copy number in cells treated with the indicated Resveratrol concentrations. qPCR was carried out as described in [89]. C–E. Flow cytometry analysis of KLEC.219 (GFP positive) for RFP expression. Cells were treated with Resveratrol or TPA at the indicated dose and time period, or infected with the indicated lentiviruses. The numbers denote the percentage of RFP positive cells, which reflects lytic cells. F. Relative KSHV DNA copy number in 293T cells infected using the growth media of BCBL1 cell infected with the indicated lentiviruses. (TIF) [file ppat.1004400.s007.tif]
