# Supplementary material for: Kaposi's Sarcoma Herpesvirus MicroRNAs Induce Metabolic Transformation of Infected Cells
Source: PLoS Pathog. 2014 Sep 25;10(9):e1004400. doi: 10.1371/journal.ppat.1004400 (PMC4177984; doi:10.1371/journal.ppat.1004400)
Supplement: Table S1 — KSHV miRNAs that are predicted by the PITA algorithm to target EGLN2 or HSPA9 and found to down-regulate the mRNA levels of these genes or to reduce luciferase activity in 3′UTR assay (shown in Figure 3C and D ). (PDF) [file ppat.1004400.s008.pdf]

Table S1

Gene	microRNA	Position	mRNA down regulation (figure 3C)	Reduced luciferase activity (figure 3D)
EGLN2	kshv-mir-k12-6-3p	1510	+	+
EGLN2	kshv-mir-k12-6-5p	346	+	+
EGLN2	kshv-mir-k12-8	511	+	+
EGLN2	kshv-mir-k12-3	451	+	+
EGLN2	kshv-mir-k12-7	454	-	+
EGLN2	kshv-mir-k12-8	430	+	+
EGLN2	kshv-mir-k12-8	1561	+	+
EGLN2	kshv-mir-k12-6-5p	640	+	+
EGLN2	kshv-mir-k12-11	1521	+	+
EGLN2	kshv-mir-k12-9	1566	-	-
EGLN2	kshv-mir-k12-7	1062	-	+
EGLN2	kshv-mir-k12-6-3p	1457	+	+
HSPA9	kshv-mir-k12-11	3210	+	+
HSPA9	kshv-mir-k12-4-3p	2458	+	-
HSPA9	kshv-mir-k12-4-3p	2436	+	-
HSPA9	kshv-mir-k12-3	1609	+	+
