# Supplementary material for: Kaposi's Sarcoma Herpesvirus MicroRNAs Induce Metabolic Transformation of Infected Cells
Source: PLoS Pathog. 2014 Sep 25;10(9):e1004400. doi: 10.1371/journal.ppat.1004400 (PMC4177984; doi:10.1371/journal.ppat.1004400)
Supplement: Table S2 — Cellular miRNAs which are predicted to target EGLN2 according to the algorithm miRror [83] . (PDF) [file ppat.1004400.s009.pdf]

Table S2

targets	miRIS	p-value	DB number	miR number	MAMI	PITA_TOP	PicTar_4way	RNA22	TargetRank_all	TargetScan_Con	miRDB	miRNAMap2	microCosm	microRNA.org_Con	microT	mirZ
hsa-let-7a	0.583333333	0.005167709	2	2	-	X	X	-	-	0.005167709	-	-	-	-	X	0.015076521
hsa-let-7b	0.583333333	0.005167709	2	2	-	X	X	-	-	0.005167709	-	-	-	-	X	0.015076521
hsa-let-7c	0.583333333	0.005167709	2	2	-	X	X	-	-	0.005167709	-	-	-	-	X	0.015076521
hsa-let-7e	0.583333333	0.005167709	2	2	-	X	X	-	-	0.005167709	-	-	-	-	X	0.015076521
hsa-let-7f	0.583333333	0.005167709	2	2	-	X	X	-	-	0.005167709	-	-	-	-	X	0.015076521
hsa-let-7g	0.583333333	0.005167709	2	2	-	X	X	-	-	0.005167709	-	-	-	-	X	0.015076521
hsa-let-7i	0.583333333	0.005167709	2	2	-	X	X	-	-	0.005167709	-	-	-	-	X	0.015076521
hsa-miR-103	0.583333333	0.007581338	2	2	-	X	0.007581338	-	-	-	-	-	-	-	X	0.04947369
hsa-miR-107	0.583333333	0.007738576	2	2	-	X	0.007738576	-	-	-	-	-	-	-	X	0.04947369
hsa-miR-1202	0.583333333	0.000403914	2	2	-	X	-	-	-	0.000403914	-	-	-	-	-	0.023484485
hsa-miR-1301	0.625	0.000411296	3	2	-	X	-	-	-	0.000686071	0.000411296	-	-	-	-	0.003696582
hsa-miR-15a	0.625	0.007716015	3	2	-	X	0.007716015	-	-	0.007751323	-	-	-	-	X	0.044724581
hsa-miR-15b	0.625	0.007751323	3	2	-	X	0.007874637	-	-	0.007751323	-	-	-	-	X	0.044724581
hsa-miR-16	0.625	0.007626098	3	2	-	X	0.007626098	-	-	0.007751323	-	-	-	-	X	0.044724581
hsa-miR-195	0.625	0.007751323	3	2	-	X	0.007783798	-	-	0.007751323	-	-	-	-	X	0.044724581
hsa-miR-2110	0.583333333	0.000270006	2	2	-	-	-	-	-	0.000270006	-	-	-	-	-	0.041187155
hsa-miR-23a	0.625	0.003630087	3	2	-	X	0.003630087	-	-	0.006028147	-	-	-	-	X	0.041229075
hsa-miR-23b	0.625	0.003661081	3	2	-	X	0.003661081	-	-	0.006028147	-	-	-	-	X	0.041229075
hsa-miR-23c	0.583333333	0.006028147	2	2	-	-	-	-	-	0.006028147	-	-	-	-	-	0.041229075
hsa-miR-3150a-3p	0.583333333	0.000214377	2	2	-	-	-	-	-	0.000214377	0.000530276	-	-	-	-	-
hsa-miR-326	0.583333333	0.001618529	2	2	-	X	0.001618529	-	-	-	-	-	-	-	X	0.045191637
hsa-miR-346	0.583333333	0.000545214	2	2	-	X	X	-	0.000545214	-	-	-	-	-	X	0.013211012
hsa-miR-3652	0.583333333	0.000532748	2	2	-	-	-	-	-	0.000532748	-	-	-	-	-	0.036983521
hsa-miR-3660	0.583333333	0.000347761	2	2	-	-	-	-	-	0.000347761	-	-	-	-	-	0.027071968
hsa-miR-3921	0.583333333	0.000740708	2	2	-	-	-	-	-	0.000740708	-	-	-	-	-	0.025224496
hsa-miR-3944-5p	0.583333333	5.91E-05	2	2	-	-	-	-	-	0.000464266	5.91E-05	-	-	-	-	-
hsa-miR-4268	0.583333333	0.000205728	2	2	-	-	-	-	-	0.000205728	-	-	-	-	-	0.022918299
hsa-miR-4290	0.583333333	0.00025075	2	2	-	-	-	-	-	0.00025075	-	-	-	-	-	0.023242487
hsa-miR-455-3p	0.583333333	0.000456948	2	2	-	X	-	-	-	0.000456948	-	-	-	-	X	0.017011281
hsa-miR-4660	0.583333333	0.00011131	2	2	-	-	-	-	-	0.000695031	0.00011131	-	-	-	-	-
hsa-miR-5047	0.583333333	0.000406666	2	2	-	-	-	-	-	0.000686071	0.000406666	-	-	-	-	-
hsa-miR-545	0.583333333	0.006147437	2	2	-	X	-	-	0.006147437	-	-	-	-	-	X	0.046251384
hsa-miR-765	0.583333333	0.000455127	2	2	-	X	-	-	0.003425318	0.000455127	-	-	-	-	X	X
hsa-miR-939	0.583333333	0.000180852	2	2	-	X	-	-	-	0.000180852	-	-	-	-	X	0.044710025
hsa-miR-98	0.583333333	0.005167709	2	2	-	X	X	-	-	0.005167709	-	-	-	-	X	0.015076521
