# Supplementary material for: Kaposi's Sarcoma Herpesvirus MicroRNAs Induce Metabolic Transformation of Infected Cells
Source: PLoS Pathog. 2014 Sep 25;10(9):e1004400. doi: 10.1371/journal.ppat.1004400 (PMC4177984; doi:10.1371/journal.ppat.1004400)
Supplement: Table S3 — Cellular miRNAs which are predicted to target all three HIF prolyl hydroxylase (EGLN1-3) according to the algorithm miRror [83] . (PDF) [file ppat.1004400.s010.pdf]

Table S3

targets	miRIS	p-value	DB number	miR number	MAMI	PITA_TOP	PicTar_4way	RNA22	TargetRank_all	TargetScan_Con	miRDB	miRNAMap2	microCosm	microRNA.org_Con	microT	mirZ
hsa-miR-106a	0.416666667	0.019238648	2	2	-	X	-	-	0.020641682	0.019238648	-	-	-	-	X	X
hsa-miR-106b	0.416666667	0.019238648	2	2	-	X	X	-	0.020641682	0.019238648	-	-	-	-	X	X
hsa-miR-17	0.416666667	0.019238648	2	2	-	X	-	-	0.020641682	0.019238648	-	-	-	-	X	X
hsa-miR-20a	0.416666667	0.019238648	2	2	-	X	X	-	0.020641682	0.019238648	-	-	-	-	X	X
hsa-miR-20b	0.416666667	0.019238648	2	2	-	X	X	-	0.020641682	0.019238648	-	-	-	-	X	X
hsa-miR-3126-3p	0.416666667	0.000276701	2	2	-	-	-	-	-	-	0.000276701	-	-	-	-	0.049278021
hsa-miR-3128	0.416666667	0.000553185	2	2	-	-	-	-	-	0.001614342	0.000553185	-	-	-	-	-
hsa-miR-346	0.666666667	0.001517859	4	3	-	X	X	-	0.001610288	-	-	-	-	0.008616857	0.047545634	0.001517859
hsa-miR-424	0.625	0.016282592	3	3	-	X	-	-	-	0.021889996	-	-	-	-	0.017823325	0.016282592
hsa-miR-429	0.416666667	0.006982186	2	2	-	X	-	-	0.020895104	-	0.006982186	-	-	-	X	X
hsa-miR-497	0.625	0.016282592	3	3	-	X	-	-	-	0.021889996	-	-	-	-	0.017466869	0.016282592
hsa-miR-519d	0.458333333	0.007262511	3	2	-	X	-	-	0.020641682	0.019238648	0.007262511	-	-	-	X	X
hsa-miR-545	0.583333333	0.009945011	2	3	-	X	-	-	0.017479471	-	-	-	-	-	X	0.009945011
hsa-miR-93	0.416666667	0.019238648	2	2	-	X	X	-	0.020641682	0.019238648	-	-	-	-	X	X
