# Supplementary material for: Kaposi's Sarcoma Herpesvirus MicroRNAs Induce Metabolic Transformation of Infected Cells
Source: PLoS Pathog. 2014 Sep 25;10(9):e1004400. doi: 10.1371/journal.ppat.1004400 (PMC4177984; doi:10.1371/journal.ppat.1004400)
Supplement: Table S4 — List of primers used in this study. (PDF) [file ppat.1004400.s011.pdf]

Table S4

		Forward	Reverse	Probe	comments
1	HSPA9	GTATTCTCTACTGCCGCTGATG	TTCAATCTGAGGAACTCCACG		qRT-PCR
2	EGLN2	GGTTGCTCATTTTCTGGTCTG	CACCTTTCTGTCCTGATGCTAG		qRT-PCR
3	TOMM40	GCA CTG TCA TGT CTC TAG CTG	CCT CAA ACT CCA CAC CCA C		qRT-PCR
4	TIMM23	TCG GCT AGG ATT GAA GGA AAC	GAT GAC ACC AAA TGC ACT ATA GAG		qRT-PCR
5	TIMM22	GCC ATG TTT TCT TGT ACT GAG TG	GCA AAA CCT CCA CAA CCA ATG		qRT-PCR
6	TUBB	ATG GAC GAG ATG GAG TTC	TTG AGT AAG ACG GCT AAG G		qRT-PCR
7	TOMM22	GAT TGT TAT TGC TGT TTG AGC TG	CAC AGT GTC ATT ATA GAC TAA AG		qRT-PCR
8	TIMM8B	TGTAGACCGCTTCATTGACAC	CCCCACTGACCCTTAAATCTG		qRT-PCR
9	TIMM13	TGGAAATGAACAGGGTGGG	ACAACTCCGAGCAGAAGT		qRT-PCR
10	TOMM20	AGAGAAGCTTGCCAAGGAG	CTACGCCCTTCTCATATTCAC		qRT-PCR
11	ORF65	GGATGAGAGGGTTGTGAGAATG	CTCGGGAAGCAGTATAACCAC		qRT-PCR
12	K8.1	TCCCAGCAATAAACCCACAG	GTAACCGTGTGCCATTTTCTG		qRT-PCR
13	B2M	ATC ATG GAG GTT TGA AGA TGC CGC	ACC AGA TTA ACC ACA ACC ATG CCT	FAM-TAT CTG AGC AGG TTG CTC CAC AGG TA-TAM	qPCR for mitochondria copy number
14	mit tRNA	ATC ATC TCA ACT TAG TAT TAT ACC C	GTA CAA TGA GGA GTA GGA GGT TG		qPCR for mitochondria copy number
15	HSPA9 3'UTR	CTC GAG TAA TAG CAG AAA TTT TGA AGC CAG	GCG GCC GCT AGG GAA AGA AAT CTG GGT TC		For cloning into luciferase assay
16	HSPD1 3'UTR	CTC GAG TCC TAG ACT AGT GCT TTA CC	GCG GCC GCT AAC TTT AAA CAA ATT TTT ATT ACA C		For cloning into luciferase assay
17	TOMM22 3'UTR	CTC GAG TAG ATT GTT ATT GCT GTT TGA GC	GCG GCC GCC CCT ACA AAT ACT TTA TAA TTA CAC		For cloning into luciferase assay
18	TOMM40 3'UTR	CTC GAG CGG CTG AGC CCT CCT G	GCG GCC GCC TGT AAA ATG TCC CGG TTC T		For cloning into luciferase assay
19	TIM23 3'UTR	CTC GAG AGA TTT TGC CAA CTC ATG AAT GG	GCG GCC GCG GAG TTA TAA CTT TGG TTC TG		For cloning into luciferase assay
20	EGLN2 3'UTR	CTC GAG GGC CAG TCC CAG AGC C	GCG GCC GCC AGA TGT GTG CAG CTC TGA		For cloning into luciferase assay
21	mirK3 mutation	GCC TGT AAT GGG CTA TGT GAG GGT GAG GAC GGC AGC G	CGC TGC CGT CCT CAC CCT CAC ATA GCC CAT TAC AGG C		For site directed mutagenesis
22	HSPA9 Cloning	GGA TCC ATG ATA AGT GCC AGC CGA G	GCG GCC GCT TAC TGT TTT TCC TCC TTT TGA TC		For cloning into pENTR (BamHI+NotI)
23	HSPA9 Flag	GGA TCC ATG ATA AGT GCC AGC CGA G	GCG GCC GCT TAc tta tcg tcg tca tcc ttg taa tcC TGT TTT TCC TCC TTT TGA TC		For cloning into pENTR (BamHI+NotI)
